# Associations between microbial communities and key chemical constituents in U.S. domestic moist snuff

**DOI:** 10.1371/journal.pone.0267104

**Published:** 2022-05-04

**Authors:** Robert E. Tyx, Angel J. Rivera, Glen A. Satten, Lisa M. Keong, Peter Kuklenyik, Grace E. Lee, Tameka S. Lawler, Jacob B. Kimbrell, Stephen B. Stanfill, Liza Valentin-Blasini, Clifford H. Watson

**Affiliations:** 1 Centers for Disease Control and Prevention, National Center for Environmental Health, Division of Laboratory Sciences, Atlanta, Georgia, United States of America; 2 Centers for Disease Control and Prevention, National Center for Chronic Disease Prevention and Health Promotion, Division of Reproductive Health, Atlanta, Georgia, United States of America; 3 Department of Gynecology and Obstetrics, Emory University School of Medicine, Atlanta, Georgia, United States of America; 4 Battelle Analytical Services, Atlanta, Georgia, United States of America; 5 Gangarosa Department of Environmental Health, Rollins School of Public Health, Emory University, Atlanta, Georgia, United States of America; University of Illinois Urbana-Champaign, UNITED STATES

## Abstract

**Background:**

Smokeless tobacco (ST) products are widely used throughout the world and contribute to morbidity and mortality in users through an increased risk of cancers and oral diseases. Bacterial populations in ST contribute to taste, but their presence can also create carcinogenic, Tobacco-Specific *N*-nitrosamines (TSNAs). Previous studies of microbial communities in tobacco products lacked chemistry data (e.g. nicotine, TSNAs) to characterize the products and identify associations between carcinogen levels and taxonomic groups. This study uses statistical analysis to identify potential associations between microbial and chemical constituents in moist snuff products.

**Methods:**

We quantitatively analyzed 38 smokeless tobacco products for TSNAs using liquid chromatography with tandem mass spectrometry (LC-MS/MS), and nicotine using gas chromatography with mass spectrometry (GC-MS). Moisture content determinations (by weight loss on drying), and pH measurements were also performed. We used 16S rRNA gene sequencing to characterize the microbial composition, and additionally measured total 16S bacterial counts using a quantitative PCR assay.

**Results:**

Our findings link chemical constituents to their associated bacterial populations. We found core taxonomic groups often varied between manufacturers. When manufacturer and flavor were controlled for as confounding variables, the genus *Lactobacillus* was found to be positively associated with TSNAs. while the genera *Enteractinococcus* and *Brevibacterium* were negatively associated. Three genera (*Corynebacterium*, *Brachybacterium*, and *Xanthomonas*) were found to be negatively associated with nicotine concentrations. Associations were also investigated separately for products from each manufacturer. Products from one manufacturer had a positive association between TSNAs and bacteria in the genus *Marinilactibacillus*. Additionally, we found that TSNA levels in many products were lower compared with previously published chemical surveys. Finally, we observed consistent results when either relative or absolute abundance data were analyzed, while results from analyses of log-ratio-transformed abundances were divergent.

## Introduction

Smokeless tobacco (ST) use contributes to oral diseases, increases cancer risks, and results in an unnecessary burden on the healthcare system [1, 2]. Moist snuff is the largest category of smokeless tobacco products sold in the United States, having an estimated 5.9 million users [3]. The negative effects of ST are attributed to the wide range of toxicants contained within each product. The microbial components of ST impact its chemistry through agricultural practices, curing, and manufacturing steps. These processes range from the steps of curing, through aging and fermentation, all of which contribute to the product’s palatability. These processes create a metabolically active environment [4–6] that incidentally results in more harmful products [7, 8].

During the tobacco curing and aging, nitrate-reducing microorganisms convert nitrate (NO_3_^-^) to nitrite (NO_2_^-^) [9]. Nitrite is a reactive species known to be actively transported out of the cells in some bacterial species [10, 11]. Once nitrite is in the extracellular environment, it reacts abiotically with abundant tobacco alkaloids, such as nicotine and nornicotine, that have been released by ruptured cells, forming Tobacco-Specific *N*-Nitrosamines (TSNAs). These chemical reactions occur more favorably at the low pH conditions during curing and aging of tobacco [4, 8, 12, 13]. TSNAs are some of the most potent and abundant carcinogens in smokeless tobacco. Two TSNA compounds in particular, *N*’-Nitrosonornicotine (NNN) and 4-(Methylnitrosamino)-1-(3-pyridyl)-1-butanone (NNK), have been identified by the International Agency for the Research on Cancer (IARC) as Group I carcinogens (known human carcinogens) [14]. Various means have been suggested to reduce TSNAs in ST products. These include sanitizing fermentation vats, adding non-nitrate reducing bacteria [5], and using agents such as green tea extract or ascorbic acid to neutralize nitrite [15]. Additionally, seeding of a microbe identified as a nitrite-reducing strain of *Bacillus amyloliquefaciens* to scavenge nitrite has been suggested [16]. Some microbial nitrate reduction has been achieved by newer farming and manufacturing techniques [17]. As microbial activity remains a key process in domestic smokeless tobacco manufacturing, TSNA formation may not be significantly reduced without a fundamental change such as the pasteurization of selected Swedish snus products [18].

The microbial taxa responsible for nitrate-to-nitrite conversion in smokeless tobacco are not known. Culture-independent studies [19–24] have confirmed the presence of diverse bacterial communities in ST products, but fail to yield definitive answers as to what microbes may be associated with TSNAs. Most microbial-focused studies to date investigated multiple types of products, but only included a limited number of samples that were not accompanied by relevant chemical measurements.

Several extensive chemical profiles of smokeless tobacco products including TSNA measurements have been published, but without microbial community data [25–32]. Associations between chemical attributes and microbial taxa have been studied, but characterizations are limited to fermenting tobacco intended for cigars, and in another study, lab-produced (non-commercial) smokeless products [22, 33]. Additional studies that included chemistry measurements were performed with cigarette and small cigar tobacco, where microbial community changes due to storage conditions were also explored [34–36]. However, smokeless tobacco product microbiotas are substantially different from cigar and cigarette tobacco [33, 35, 37, 38].

Although products such as snus and new “tobacco-free” nicotine pouches are rapidly gaining popularity, moist snuff products remain very popular among ST users. For instance, the moist snuff products Copenhagen, Grizzly, and Skoal were the top 3 selling ST brands on the market in 2019 (data from https://www.statista.com/). Due to their popularity, we focused on traditional moist snuff products that are fermented rather than pasteurized. Moist snuff products that utilize fermentation are clearly distinct from unfermented tobacco products, such as Swedish snus, which is subjected to heat treatment to remove microbes, thereby omitting the fermentation process [39].

This study provides an updated survey of chemistry in popular moist snuff products on the domestic market and explores microbes associated with TSNAs in these products. To examine this association we analyzed 38 smokeless tobacco products using analytical chemistry measurements (TSNAs, nicotine, pH, and moisture) and 16S microbial community surveys. Combining data from chemistry and the microbiota allowed us to relate several bacterial taxa with TSNAs. Since both the relative and absolute abundance of taxa were measured, this study also provides an opportunity to compare and contrast the results of analyses of these different data types, as well as analyses based on log-ratios of abundance data.

## Materials and methods

### Samples, preparation and storage

Commercial smokeless tobacco products (N = 38) were purchased in 2016 by Lab Depot (Dawsonville, GA, USA) and shipped to CDC. Moisture samples were taken from individual tins, then three packages, or tins, of each tobacco product were pooled to ensure complete homogenization. After moisture measurement aliquots were taken, the remaining contents were placed into large polypropylene tubes and rotated for 30 minutes. Samples were stored at -80 °C until thawed for DNA extraction. Aliquots were taken for chemistry after thawing and prior to taking samples for the microbiological experiments.

We focused on moist snuff, but also included one product, Hawken Wintergreen (Hawken), that is marketed towards moist snuff users, but is substantially different. Hawken represents a product that is compositionally more similar to a chewing tobacco. Hawken has generally been viewed as a “introductory” product with lower pH and nicotine, which would deliver less free nicotine during use, presumably alleviating nausea caused by high nicotine levels [40].

Two versions of Copenhagen Long Cut Straight with different labeling were obtained, which we termed ‘A’ and ‘B’. Aside from the labeling on the package, contents of the two versions appeared identical.

All quantitative analytical chemistry measurements were performed in accordance with laboratory ISO 17025 quality guidelines. After aliquoting for chemical measurements, samples were stored at -20 °C prior to measurement and allowed to equilibrate to room temperature before analysis.

### Quantifications of moisture and pH

Moisture was quantified by the mass loss on drying method described in Lawler *et al*., 2013 [32]. Moisture was measured with two replicates for each product and the means are presented in the results. The weight difference of freshly opened product (prior to pooling) versus dried tobacco was used to determine moisture. Tobacco was dried at 99 °C for 3 hours and then placed in a desiccator for approximately 30 minutes [41].

The product pH was measured as previously described in Lawler, *et al*., 2013 [32, 41]. Briefly, 10 mL of deionized distilled water was added to 1.0 g of sample and measured on Sirius Vinotrate pH meters (Sirius Analytical, East Sussex, UK). The meter was calibrated daily with pH buffers of 4.01, 7.00, and 10.01. Duplicate pH readings were averaged.

### Quantification of nicotine by gas chromatography with mass spectrometry and free nicotine calculations

Nicotine was extracted from ST products and subsequently analyzed, in triplicate, using an Agilent 6890 Gas Chromatograph/5973N Mass Spectrometer fitted with an Agilent Ultra2 GC column (25 m x 0.32 mm x 0.52 μM) (Agilent Technologies; Santa Clara, CA) with parameters described elsewhere [42]. Methyl *tert*-butyl ether (MTBE), sodium hydroxide (NaOH) and chemical standard quinoline were purchased from Sigma-Aldrich (St. Louis, MO). Nicotine standards were obtained from Accustandard (New Haven, CT, USA). Briefly, the method involves weighing a 0.4 g product sample into a sample bottle then adding 1 mL of 2N NaOH and 10 mL MTBE with quinoline added as an internal standard. The extraction solution plus sample were agitated on a Rugged Rotator for 60 minutes at 70 rpm. Approx. 1.5 ml extract was transferred to a 2 mL autosampler vial and a 1-μL aliquot of each sample extract was injected into the GC/MS operated in selected ion monitoring mode. GC parameters included: column flow rate of 1.7 mL/minute and an inlet temperature of 230 °C; and the auxiliary line temp was held at 280 °C. The GC oven ramp parameters were as follows: hold 175 °C for 1 min; ramp at 5 °C/minute to 180 °C; and finally, ramp at 35 °C/minute to 240 °C. The total run time is 3.7 minutes. Relative response factors (nicotine quantitation ion area/quinoline quantitation ion area) against nicotine concentrations resulted in a calibration curve that was used to quantify total nicotine.

Unprotonated (free or freebase) nicotine is the charge neutral form of nicotine that is most easily released from tobacco and absorbed across oral membranes. Free nicotine percentage was calculated using the measured pH of the product and the pK_a_ value of the pyrrolic nitrogen of nicotine (8.02) substituted into the Henderson-Hasselbach equation [41]. The percentage of free nicotine was multiplied by total nicotine to get the amount of free nicotine (mg/g).

### Quantification of Tobacco-Specific Nitrosamines (TSNAs) by liquid chromatography with mass spectrometry

Five tobacco-specific-*N*’-nitrosamine compounds were analyzed: *N*’-nitrosonornicotine (NNN), 4-(methylnitrosamino)-1-(3-pyridyl)-1-butanone (NNK), *N*’-nirosoanatabine (NAT), *N*’-nitrosoanabasine (NAB), and 4-(methylnitros-amino)-1-(3-pyridyl)-1-butanol (NNAL). Three replicates of tobacco samples were spiked with ^13^C labeled internal standards, then extracted with ammonium acetate buffer. The extracts were analyzed by LC-MS/MS using an Agilent 1200 (Agilent Corp., Santa Barbara, CA) equipped with a Waters XBridge MS C18 50x4.6mm 5-μm pore size column (Waters Corp., Milford, Massachusetts) and a Sciex API-4000 (Sciex Corp., Framingham, MA) tandem mass spectrometer. Eluent solvents were 5 mM ammonium acetate solution as aqueous phase and 5 mM ammonium acetate in a mixture of 95% acetonitrile and 5% water for organic phase Quantitation was done using a calibration curve with an 1/x weighting generated using the peak area ratio of analyte to labeled internal standard. The measured TSNAs were reported in ng per gram of tobacco.

### Nucleic acid extractions

Nucleic acids were extracted from tobacco products using the PowerSoil DNA Elution Accessory Kit together with the Total RNA Isolation Kit(MoBio, Carlsbad, CA, USA), with a few modifications. These kits were used for co-extraction of nucleic acids in this study because we originally intended to sequence cDNA made from extracted RNA as well as the DNA itself. We found, however, that RNA amounts that were extracted were highly variable, potentially making analysis difficult, and thus, we limited this study to an examination of the DNA. For reference, a table with RNA extraction values for the first eight products extractions are provided in S1 Table in S4 File. Extraction protocol modifications included the use of 0.5 grams tobacco, weighed into polypropylene tubes and the addition of 0.5 mL of molecular biology grade, nuclease-free water prior to extraction. Additionally, MPBio’s Lysing matrix E (MP Biomedicals, Santa Ana, CA, USA) was used *in lieu* of the bead-beating tubes provided with that kit. All bead-beating was performed using a SPEX GenoGrinder with 4 cycles of 2 minutes at 1750 RPM followed by cooling on ice for 2 minutes between grinding steps (Spex Sample Prep, Metuchen, NJ, USA). Due to varying amounts of potentially inhibitory contamination that gave eluants a varying shade of color (likely from excess humic acids), we used an additional cleanup step for further purification. This clean-up step was performed after the extraction using Qiagen DNEasy columns using the Qiagen QIAmp DNA mini kit (Qiagen, Germantown, MD, USA). All products were homogenized, then treated with RNAProtect prior to extraction (Qiagen, Germantown, MD, USA). Duplicate samples for each product were extracted and sequenced. S2 Table in S4 File lists extracted amounts of DNA for each sample.

### Library preparation and sequencing

Libraries were prepared from amplicons using primers derived from Illumina MiSeq 16S protocol with some changes, as described below. Primer specifics for the V4-V5 region of the 16S rRNA gene used [43] are provided in S3 Table in S4 File. Pooled and frameshifted primers were used to increase sequencing diversity [44, 45]. Multiplexing indexes were included in the primers, as were annealing sequences for Illumina sequencing. Three reverse primer sequences were used to provide greater coverage for the V4-V5 hypervariable regions. Sequencing was performed using the Illumina MiSeq Reagent Kit V2 (500 cycle) sequencing kit. Sequencing plate setup and index numbers used are given in S1 File. PCR amplification of 16S regions used KAPA HiFi HotStart 2X ready mix (Kapa Biosystems, Wilmington, MA, USA). Thermal cycler conditions were as follows: One cycle at 95 °C for 3 minutes, 25 cycles of: 98 °C for 30 seconds, 58 °C for 30 seconds, 72 °C for 30 seconds, then a 72 °C hold for 5 minutes followed by cooling and a final hold at 4 °C. For each reaction, 12.5 ng (1.5 ng / μl) of template DNA were used, with primer concentration at 5 μM. Ampure XP was then used for PCR cleanup, and then for library preparation, eight additional cycles were used with Nextera XT Indexes (5 μl each), followed by a further cleanup using AMPure XP.

Library quality was assessed using an Agilent Bioanalyzer 2100 with a High Sensitivity DNA chip (Agilent Technologies; Santa Clara, CA, USA), and quantified using a Qubit 2.0 with the Qubit dsDNA HS Assay Kit (Thermo Fisher; Waltham, MA, USA). The DNA libraries were then combined in equimolar amounts before going onto the sequencer. Sequencing was performed on an Illumina MiSeq using the MiSeq Reagent Kit V2 (500-cycle) (Illumina Inc., San Diego, CA, USA).

### Measurement of total bacterial load by qPCR

Measurements were completed as described in Al-Hebshi, 2017 [23]. Briefly, a small, well-conserved portion of 16S (1406F-1525R primer set) was used in conjunction with a control for inhibition. The inhibition control, run in parallel to the 16S samples, used samples spiked with genomic DNA extracted from DH10B *E*. *coli*. A standard curve using serial dilutions of the *rpsL* gene was constructed. Total bacterial 16S counts were computed based on the slope of the calculated calibration curve. Three dilutions of each sample were run, in triplicate. Averages of triplicates were used in calculation of the 16S counts per 1 gm of tobacco. One sample, Stoker’s Long Cut Natural, was omitted due to loss of the DNA sample prior to the qPCR bacterial load quantitation.

### Bioinformatics analysis—Data QC processing and 16S pipeline

Sequences were uploaded to National Center for Biotechnology Information (NCBI) Sequence Read Archive (SRA) with the BioProject ID PRJNA684146. FaQCs v2.08 was used to generate statistics on average read length, GC %, and average quality score per pair of reads (S4 Table in S4 File).

Paired read files were processed in QIIME2 v2019.4, [46] using dada2 denoising (version 1.8) for quality filtering and error modeling with the following parameters: trimLeft = c(23,20), truncLen = c(235,225) [47]. Taxonomy was assigned using QIIME2 naïve bayes feature-classifier “classify-sklearn” with the Silva v132 reference database [48, 49], formatted for QIIME, with classifier training using the primer set listed in S3 Table in S4 File.

The specific QIIME2 commands are listed in S2 File. An operational taxonomic unit (OTU) table was constructed after glomming taxonomy to genus level (S5 Table in S4 File), as the 16S V-region used would not accommodate useful species-level identification in all instances, yet higher level associations (e.g. family level) may not be specific enough to provide useful information on which microbes are involved. The OTU table was imported into the R statistical software suite using R package ‘qiime2R’ (https://github.com/jbisanz/qiime2R/). The R package ‘phyloseq’ was used for data visualization including alpha diversity and PCA analyses [50]. All QIIME2 commands are given in S2 File, R scripts in S3 File.

### Statistical analysis

For statistical analyses, Hawken Wintergreen was excluded from the study as it represents a different type of product (chewing tobacco vs. moist snuff); in addition, it had substantially fewer reads, many or all of which may have been artifactual, as Illumina multiplexed sequencing sometimes results in small amounts of crossover between barcodes [43]. Stoker’s LC Natural was also omitted from the statistical analysis due to a loss of the sample that prevented us from using it in the total bacterial load qPCR.

Statistical models and software packages tailored to microbiome data were used. First, we analyzed relative abundance data obtained by dividing the counts observed for each sample by the library size for that sample. We then analyzed quantitative count (absolute abundance) data obtained by multiplying the relative abundances for each sample by the measured number of 16S sequences per gram of sample. Finally, we generated centered log ratio (CLR) data by replacing abundance by its log transform, then subtracting the mean log abundance for each sample. A pseudocount of 1 was added to each zero count to allow the log to be taken. Data on all three scales were each analyzed using the R package LDM (linear decomposition model, version 4.0), first as a whole, followed by separate analyses of products from manufacturer [51]. The LDM uses statistical methods that are appropriate for the nature of microbiome data, while controlling the False Discovery Rate (FDR) [51]; we note that many other methods developed for analysis of microbiome data do not control the FDR [52].

The LDM gives both an overall (global) test of microbiome association as well as association tests for each OTU, while also allowing for control of confounding covariates. LDM was used to test for association between TSNAs, nicotine and other analytes and the microbiota, while controlling for potentially confounding factors including manufacturer, moisture, and pH. Because ‘Classic’ and ‘Crisp’ flavors were rare in our sample (see S6 Table in S4 File), we used ordination, with manufacturer as a confounder, to determine that ‘Classic’ flavor products were closest to the flavor category ‘none’ (corresponding to products where there no flavor was noted on the package); the distance between ‘classic’ and ‘none’ was smaller than the distance between any two other flavors. Thus, we assigned the products with ‘classic’ flavor the flavor value ‘none.’ Unfortunately no other flavor was close to the single ‘crisp’ product; thus, for the statistical association analyses where flavor was used as a confounder, the single “crisp” flavored product was left out of the analysis. Association between chemical analytes and individual OTUs were obtained from LDM, using a nominal false-discovery rate (FDR) of 10%. Significance was defined for LDM as q-value of less than the nominal FDR (q < 0.1) (S3 File). The direction of the identified associations were obtained using the sign of the ‘v.freq’ effects for individual taxa in the LDM (S3 File). To reduce Monte-Carlo error, we fixed the number of permutations to 1,000,000 for analyses of the entire dataset, and to 100,000 for the analyses of samples from individual manufacturers. For relative abundance analyses, p-values from the LDM omni test (that optimizes over untransformed and arc-sin root transformed tests) are reported. For the absolute abundance and CLR analyses, the arc-sin root transformation is not appropriate, so only results from the untransformed data are reported (denoted as FREQ in the LDM). The ordination plot was created using R package ‘phyloseq’ using the Bray-Curtis dissimilarity based on relative abundances. All R commands used in the analyses presented here are given in S3 File, where we also use comments to indicate how we used the LDM to analyze the quantitative count and CLR data.

## Results

### Products

All varieties of moist snuff products available and listed by the selected vendor were purchased for this study. Products that were available in different cuts were only purchased in long cut. To obtain a representative and homogenous product, with enough product for all measurements, three tins for each product were pooled together, sampled for moisture, then pooled, homogenized, and frozen for later sampling. From the pooled material, samples were taken for chemistry and microbiological measurements (S6 Table in S4 File).

### Chemical measurements and observations

**Fig 1:**
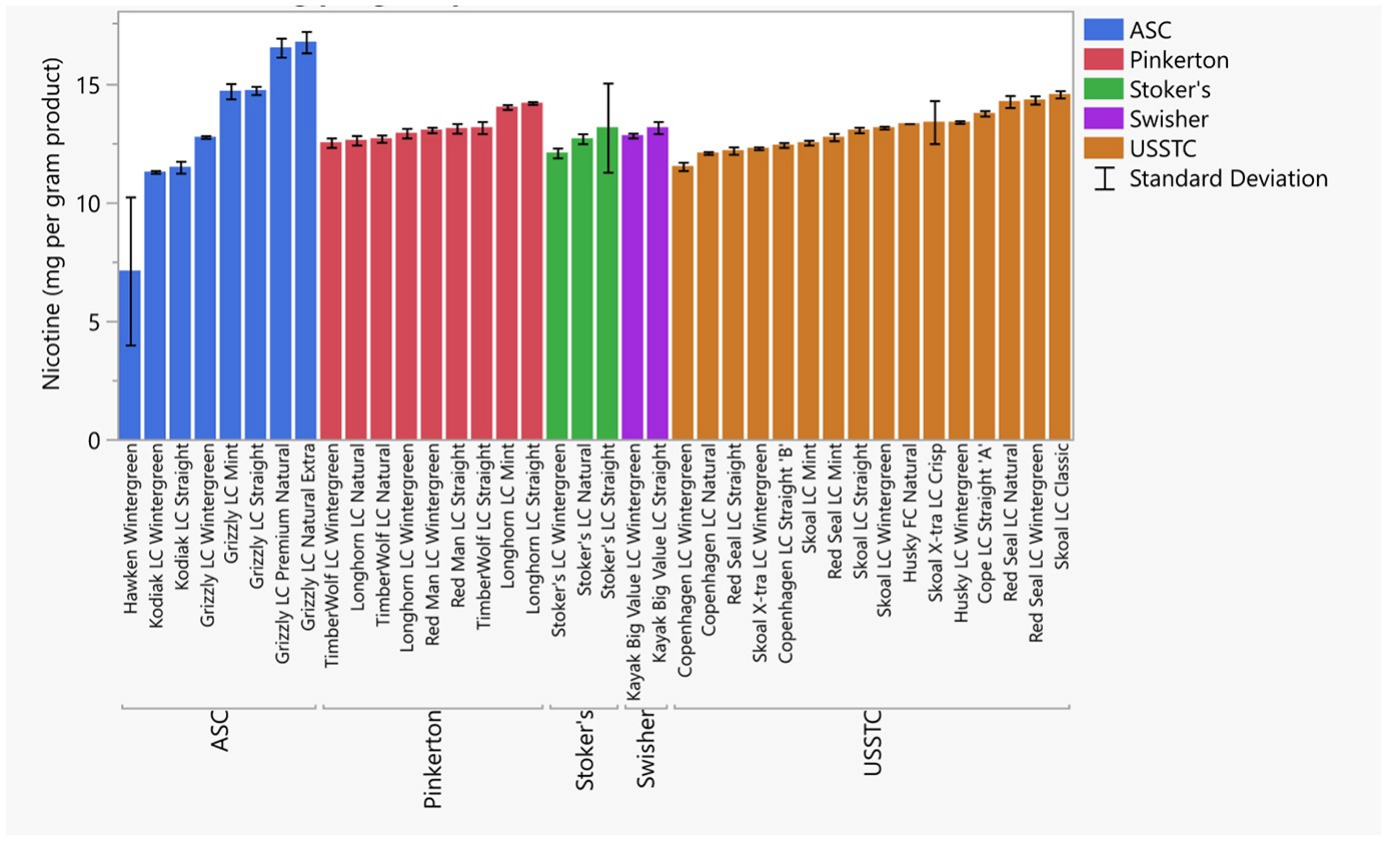
Nicotine measurements (in mg/g) by Liquid Chromatography with Mass Spectrometry (LC/MS). Error bars are constructed using one standard deviation from the mean.

**Fig 2:**
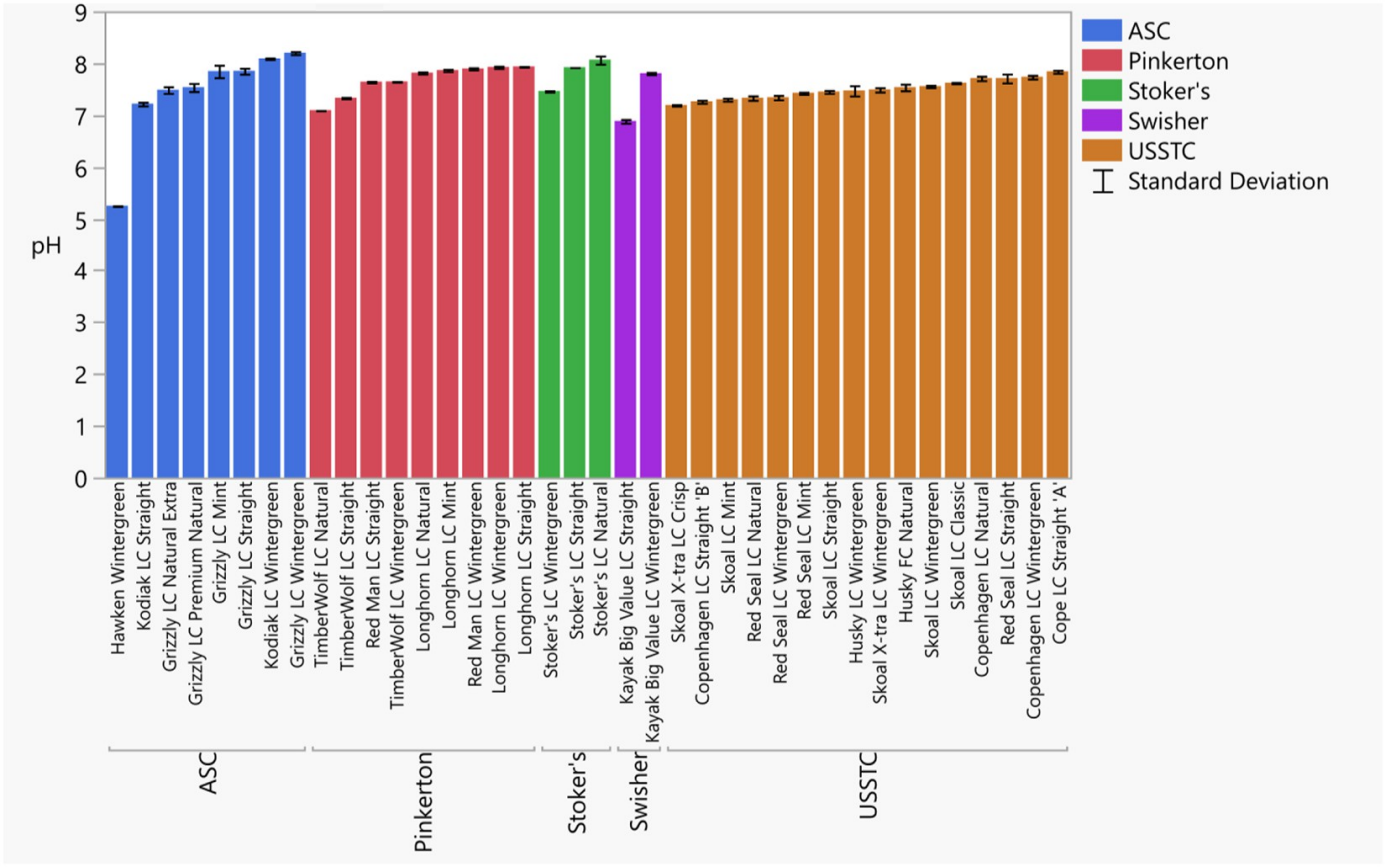
pH measurements of smokeless tobacco products. Error bars are constructed using one standard deviation from the mean.

**Fig 3:**
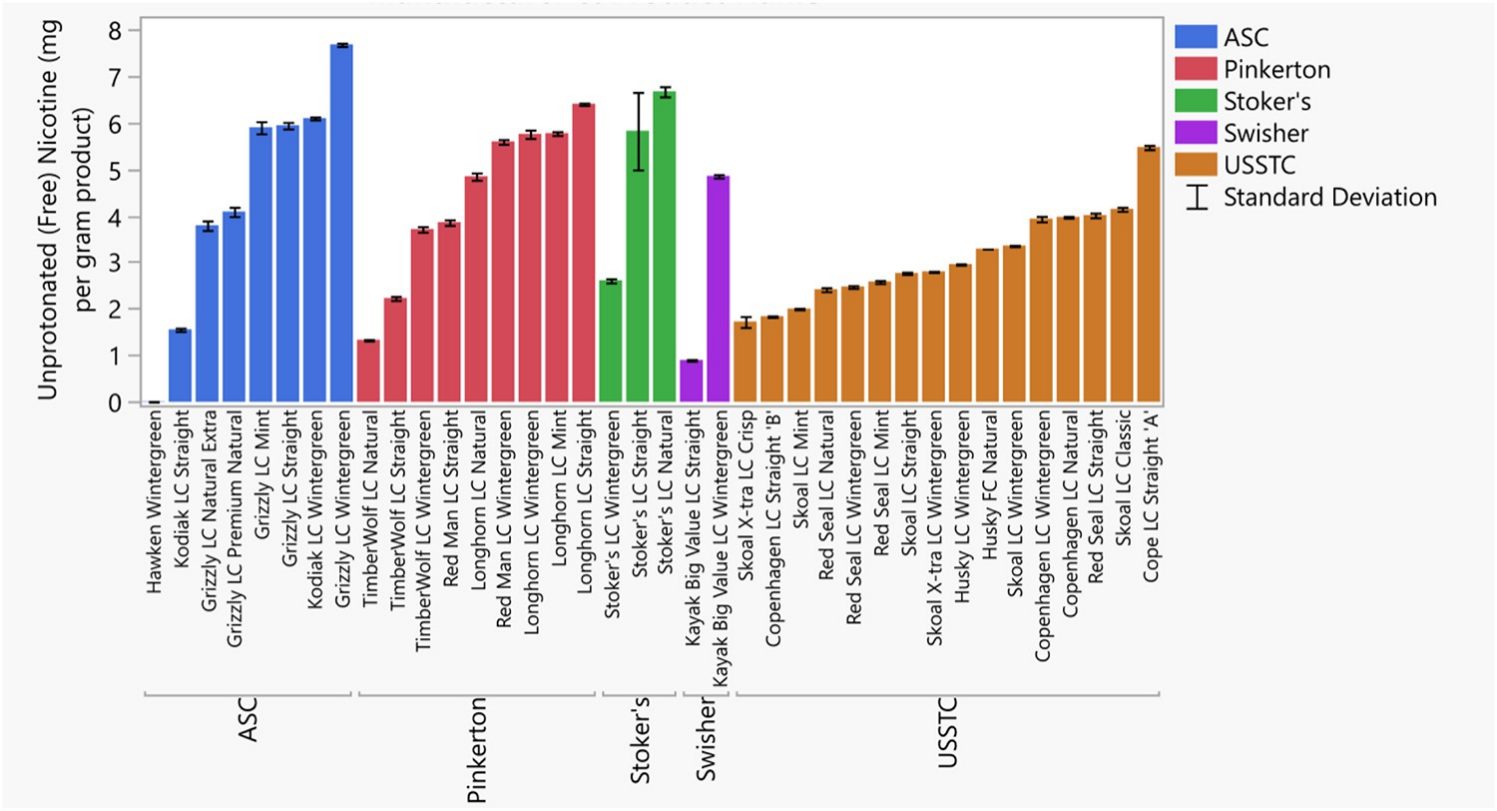
Free nicotine (in mg/g smokeless tobacco product). Free nicotine is calculated using the pKa of nicotine and the pH using the Henderson-Hasselbach equation. Error bars are constructed using one standard deviation from the mean.

Chemical measurements exhibited some variation between products with a marked differences for the product Hawken wintergreen (Hawken). For instance, the total nicotine levels of ST products ranged from 11.3 and 16.7 mg/g, while Hawken was found to be 7.10 mg/g (Fig 1, S6 Table in S4 File). Similarly, product alkalinity ranged from pH 6.89 to 8.20, yet Hawken had a pH of 5.25 (Fig 2, S6 Table in S4 File). The free nicotine ranged from 0.90 to 7.65 mg/g, with the exception of Hawken at 0.01 mg/g (Fig 3, S6 Table in S4 File). The percentage of nicotine as free nicotine in these products ranged from 6.9 to 60.2%; whereas Hawken was 0.2%. These values are consistent with past measurements of top-selling moist snuff brands [25, 53, 54].

Previously, Richter *et al*., [25], identified a correlation between the top market products and highest free nicotine. We found that the current top three market share brands, Copenhagen, Grizzly, and Skoal, had a wide range of free nicotine, from the highest of the group in Grizzly Long Cut Wintergreen (at 7.65 mg/g free nicotine) to a product with comparatively low free nicotine (Copenhagen Long Cut Straight ‘B’, at 1.84 mg/g free nicotine). The two versions of Copenhagen Long Cut Straight with different labeling were particularly interesting in that they had very different levels of free nicotine (5.45 mg/g vs 1.84 mg/g, for ‘A’ and ‘B’, respectively). We found that products with Wintergreen had significantly more free nicotine (as percentage of total nicotine, S1 Fig).

**Fig 4:**
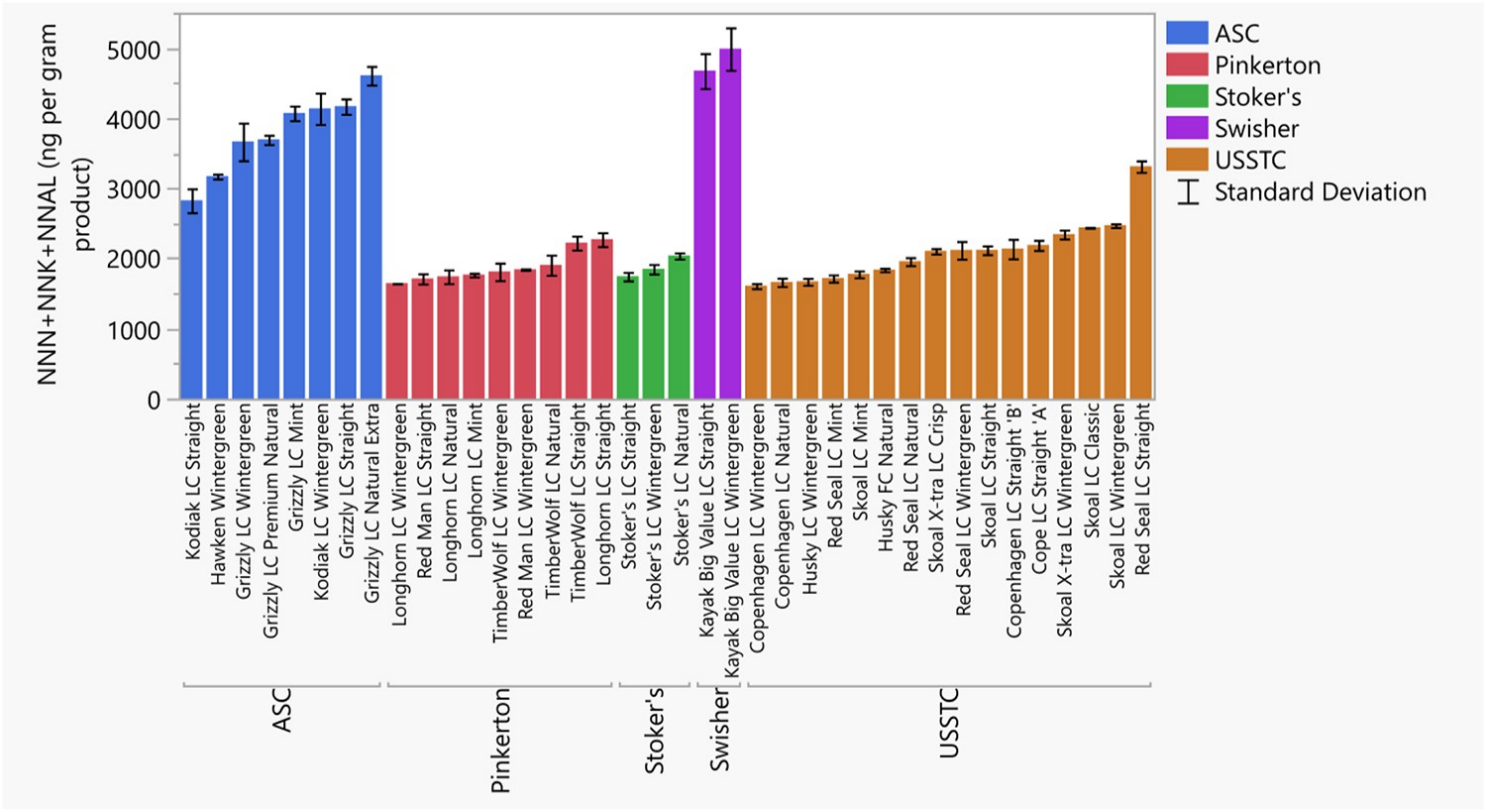
Tobacco-Specific Nitrosamines (TSNAs) measured in smokeless tobacco products. TSNAs values are given in ng/g wet-weight tobacco and are sums of three TSNAs measured: NNN, NNK, NNAL. Error bars are constructed using one standard deviation from the mean.

Measured concentrations of the carcinogenic TSNA compounds are given in Fig 4. Individual values of all TSNAs, and other information are given in S7 Table in S4 File. Values of total TSNAs (all five individual TSNA values added together) varied from 2760 to 8530 ng/g of wet weight tobacco (S7 Table in S4 File). The amounts of TSNAs varied significantly by manufacturer with Swisher and American Snuff Company (ASC) products containing the highest amounts (S2 Fig).

**Fig 5:**
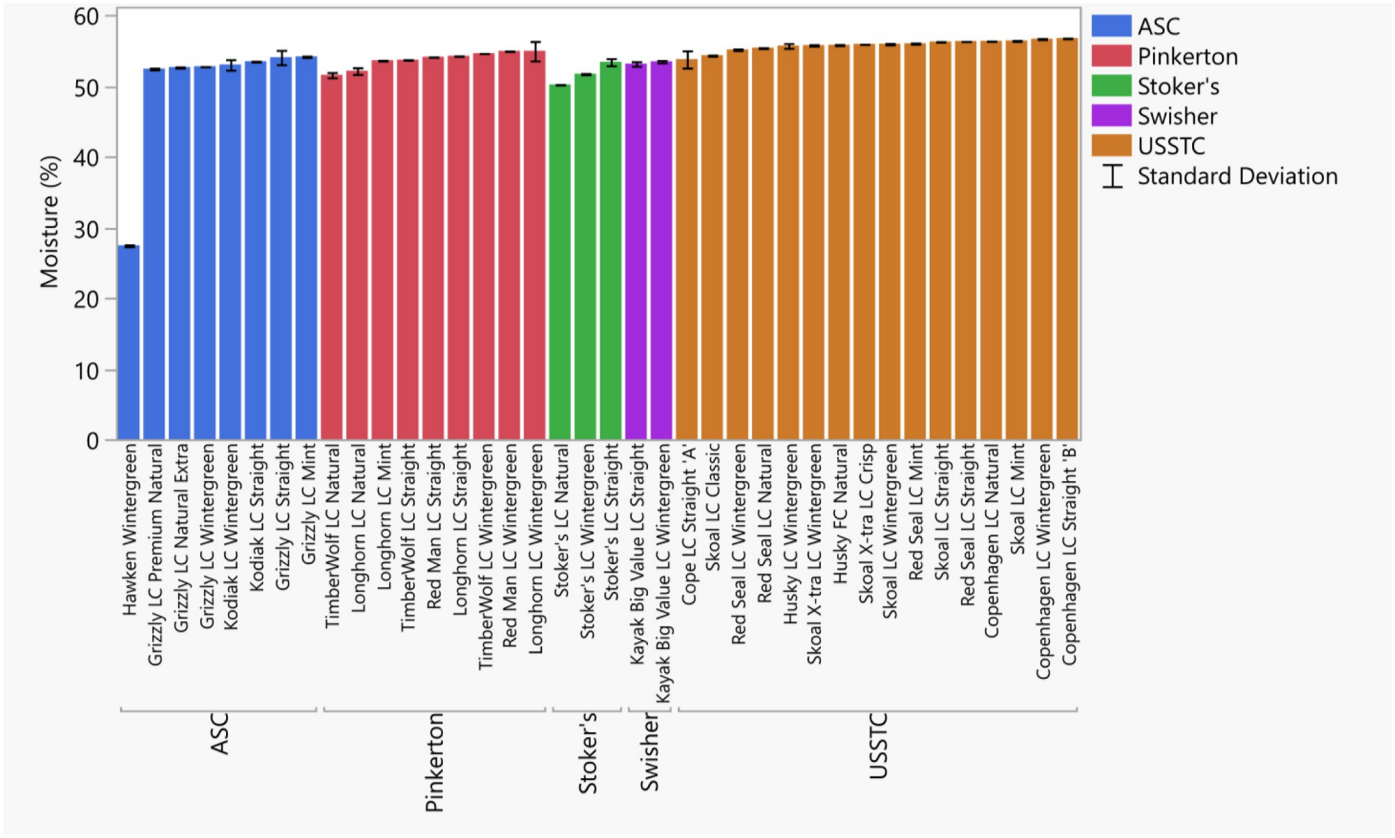
Moisture as measured by loss on drying method. Tobacco products were measured before and after a two hour incubation at 100 °C followed by cooling for 30 minutes in a desiccator. Error bars are constructed using one standard deviation from the mean.

The moisture content for most products were all just over 50% moisture by weight, with values ranging from 50.3% to 56.8% (Fig 5, S6 Table in S4 File). An exception was Hawken which had 27.4% moisture, consistent with a previous study [25]. Moisture was overall very consistent between moist products and even between our results and studies reported >10 years ago [25, 53].

A multivariate analysis of the chemistry measurements is given in S3 Fig. Aside from the expected correlation of pH with free nicotine percentage, other correlations were not observed.

### DNA extractions, bacterial load, and observations of bacterial communities

We obtained microbial community profiles for all thirty-eight products using 16S rRNA sequencing. For the main study, extraction yields were highly variable, and ranged from 0.306 to 39.2 ng/mL with an average of 9.73 ng/mL, and a standard deviation of 7.68 ng/mL (S2 Table in S4 File). Excluding Hawken Wintergreen samples, sequencing generated an average of 229,286 read pairs for each product (S4 Table in S4 File); an average of 196032 reads per sample passed QC and were classified. Hawken sequencing only averaged 4,413 read pairs, of which only 2230 passed QC and were classified. Hawken had a measurable amount of DNA extracted for each replicate (S2 Table in S4 File), but PCR amplification did not produce consistently measurable amplicons. The average read size was also shorter in Hawken (236 bases average read size vs 249 bases for the other samples). Because sequencing characteristics were not comparable to other samples, results from this product were deemed insufficient for analysis in PCA and other statistical metrics and consequently omitted from further analysis.

Total bacterial load had a median of 2.4x10^9^ 16S copies per gram of tobacco (S4 Fig). With the exception of Hawken, which had a much lower amount of bacterial load of 3.0x10^7^ copies per gram of tobacco, the range varied from 2.3x10^8^ 16S copies per gram of product (MS34) to 2x10^10^ 16S copies per gram of product (MS11), corresponding to absolute abundances that varied up to 100-fold across samples.

**Fig 6:**
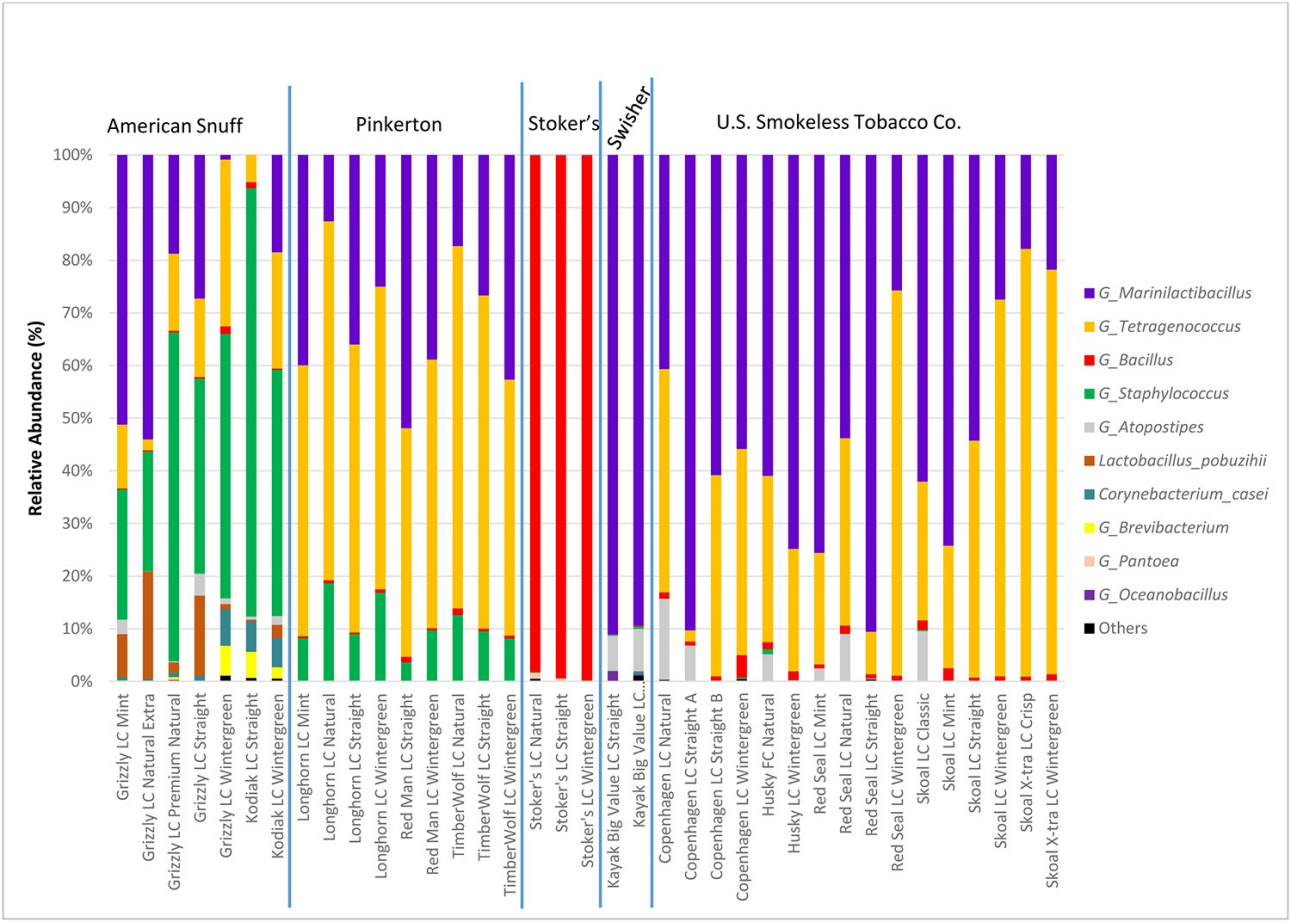
Relative abundances of top 10 Bacterial taxa in U.S. domestic moist snuff tobacco products. Bar graphs are based on number of hits for each taxonomic group, glommed by Genus. Sequencing for each sample was conducted in triplicate, with mean of three replicates presented in the figure.

**Fig 7:**
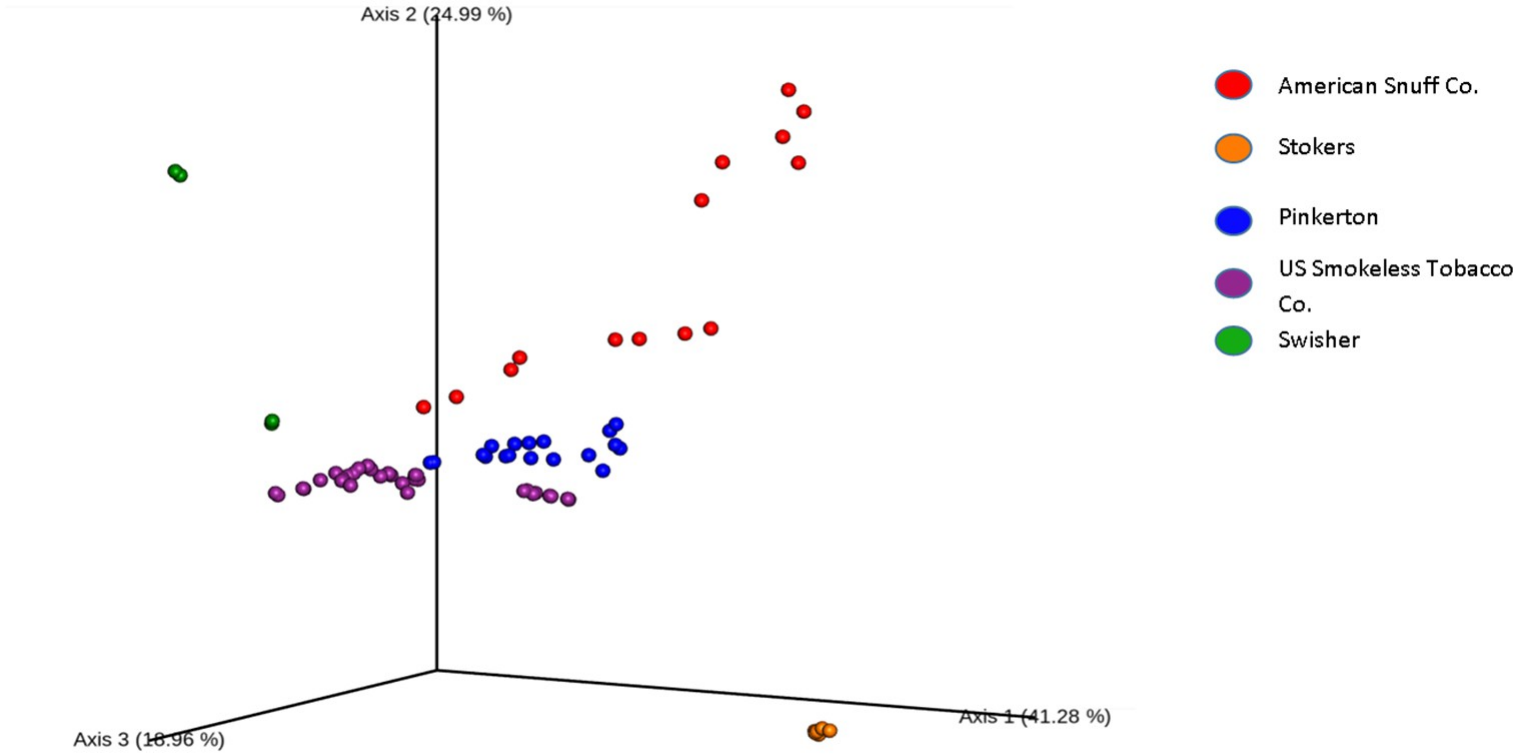
Principal Component Analysis (PCA) of moist snuff products. QIIME2 was used to generate a PCA plot representing distance between products based on Bray-Curtis values. Color represents manufacturer. Sequencing for each sample was conducted in triplicate, with mean of three replicates presented in the figure. All Stoker’s products were clustered fairly tightly together.

Qualitatively, observed bacterial communities in the moist snuff samples were, overall, fairly consistent with results obtained in previous publications based on 16S analysis [20–23]. Most samples were dominated by just a few bacterial species, mainly Firmicutes, including just a few members of the Orders Bacillales (genera including *Bacillus*, *Geobacillus*, *Oceanobacillus*, *Staphylococcus*), and Lactobacillales (genera including *Lactobacillus*, *Tetragenococcus*), and Actinobacteria (*Corynebacterium* genus). Specific taxa were brand-dependent, and relative abundances of those brand-specific taxa varied between products within the same manufacturer. Relative abundances of taxa for each product are presented in bar graph form (Fig 6). We found that overall, the greatest driver of community composition was the product manufacturer, as most of the products by a single manufacturer had similar presence or absence of taxa and clustered together in the PCA analysis (Fig 7).

### Associations between chemical analytes and the microbiome

The results of our analyses of the quantitative count data can be found in Table 1A–1C. We also analyzed these data using relative abundances and centered-log-ratio (CLR) transformed relative abundances. Data from all three scales were analyzed using the LDM. A comparison of the three results from relative, absolute and CLR-transformed abundance analyses is presented in S8 and S9 Tables in S4 File. Results for analyses using relative and absolute abundance were similar, while CLR-transformed analyses were divergent. Because absolute abundance data is thought to be the most informative, we present only these results here. Results for the other two scales can be found in S8 and S9 Tables in S4 File.

**Table 1:** Associations between product chemistry (TSNAs and nicotine) to taxa.

**A. Taxon-specific associations to the value of the sum of TSNAs NNN, NNK, NNAL** **(red or green background indicates direction of association in the LDM)** **Confounding variables: manufacturer and flavor**			
**p-value (LDM)**	**q-value (LDM)**	**Taxa**	**Direction of association**
0.0090	0.049	*Brevibacterium*	-
0.00069	0.049	*Enteractinococcus*	-
0.0015	0.024	*Lactobacillus*	+
**B. Taxon-specific associations to nicotine Confounding variables: manufacturer and flavor**			
**p-value (LDM)**	**q-value (LDM)**	**Taxa**	**Direction of association**
0.0061	0.037	*Corynebacterium*	-
0.11	0.037	*Brachybacterium*	-
0.11	0.047	*Xanthomonas*	-
**C. American Snuff Co. associations to TSNAs NNN, NNK, NNAL**			
**p-value (LDM)**	**q-value (LDM)**	**Taxa**	**Direction of association**
0.0060	0.084	*Marinilactibacillus*	-

Note: TSNAs, tobacco-specific *N*-nitrosamines; NNN, *N*’-Nitrosonornicotine; NNK, 4-(Methylnitrosamino)-1-(3-pyridyl)-1-butanone; NNAL, 4-(methylnitrosamino)-1-(3-pyridyl)-1-butanol. The Ambiguous_taxa nomenclature indicates the taxonomic group was closely related to the genus but was distant enough to not fit the criteria to glom the operation taxonomic unit (OTU) into the same genus.

We first used the LDM to determine how much of the variability found in the absolute abundance data each important variable explained. We found that Manufacturer explained 69% of the variability, while flavor, total TSNA, nicotine, moisture, and pH explained 15, 0.69, 3.0, 9.7, 2.5%, respectively. The effect of manufacturer on overall (global) taxonomic abundance was highly significant (p<0.0002). TSNAs were also significantly associated with taxa on a global level (p = 0.0064). Flavor appeared to have an effect, but when manufacturer was used as a confounder in the LDM, flavor did not reach significance as a driver of taxa globally (p = 0.61). The global association between TSNA and microbial composition was also not significant when flavor was incorporated as a confounder; however, some individual genera were found to be associated with TSNAs (Table 1A). Nicotine was not found to have a significant association with overall microbial composition on a global scale, but was also found to be associated with taxa, shown in Table 1B. Log transformations for TSNA were also investigated, but associations remained largely unchanged.

### Taxonomy highlights of samples by manufacturer

**Fig 8:**
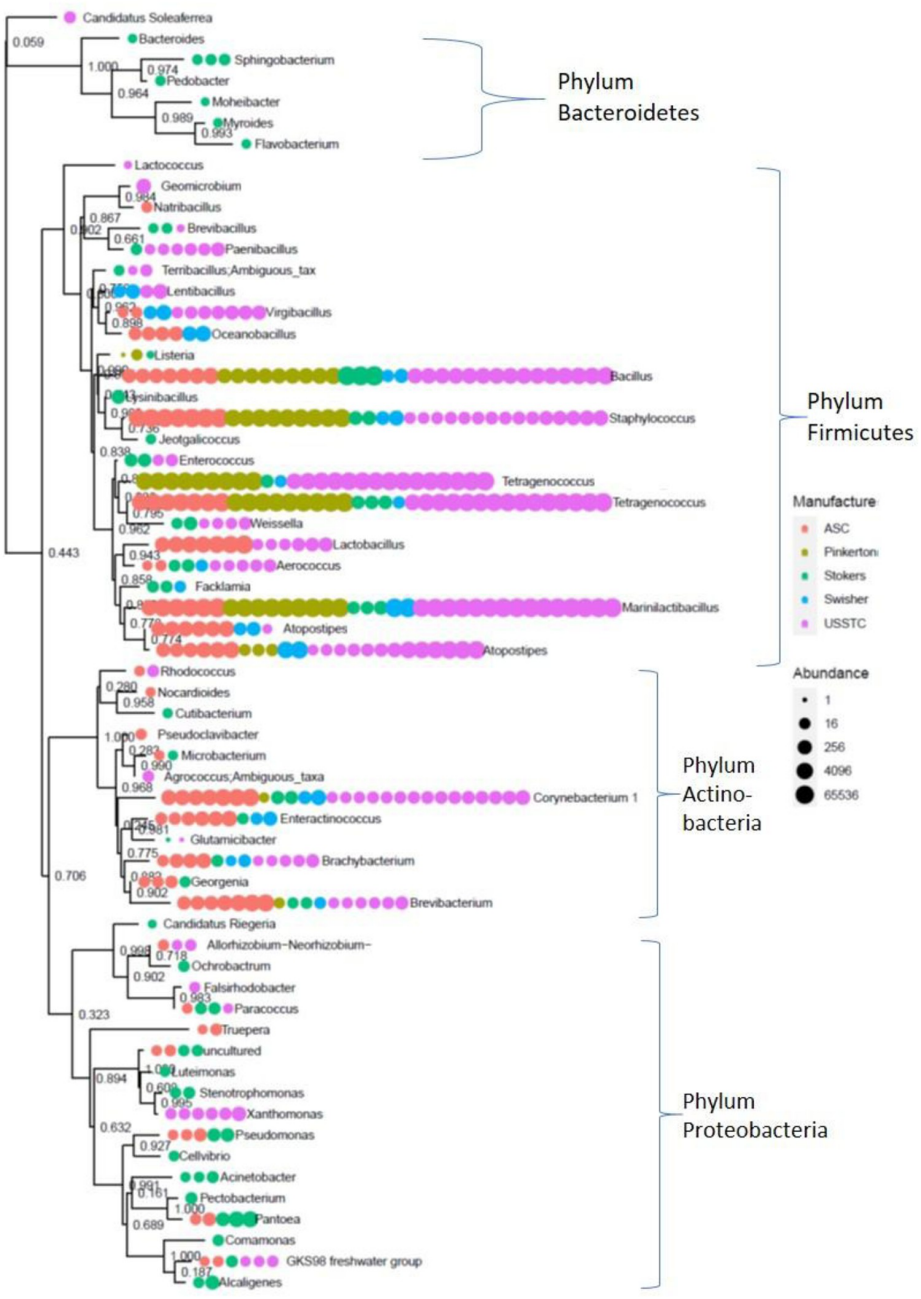
Phylogenetic tree and abundance by manufacturer. R package Phyloseq was used to generate a taxonomic tree using data glommed to Genus level. Each tip represents a Genus, with bootstrap values given at intersections. Each dot after the tree tip and label represents a product that was found to have that taxon. The size of the dots represents abundance in that particular product. Sequencing for each sample was conducted in triplicate, with mean of three replicates presented in the figure.

We also investigated associations between TSNAs and microbial taxa using the LDM with data from samples corresponding to each manufacturer individually. A phylogenetic tree with abundances colored by manufacturer is presented in Fig 8. Four phyla were represented in the samples: Actinobacteria, Bacteroidetes, Firmicutes, and Proteobacteria. In this tree, manufacturer’s microbiota and abundance patterns are demonstrated by the presence or absence of patterns found at the trees tips and sizes of the dots. For example, products manufactured by Stoker’s were unique in that their microbiota were dominated by *Bacillus* spp. with only a few other taxa present. In Fig 8, the three clades that appear at the top of the tree are comprised primarily of taxa that appear exclusively or predominantly in Stoker’s products.

**Fig 9:**
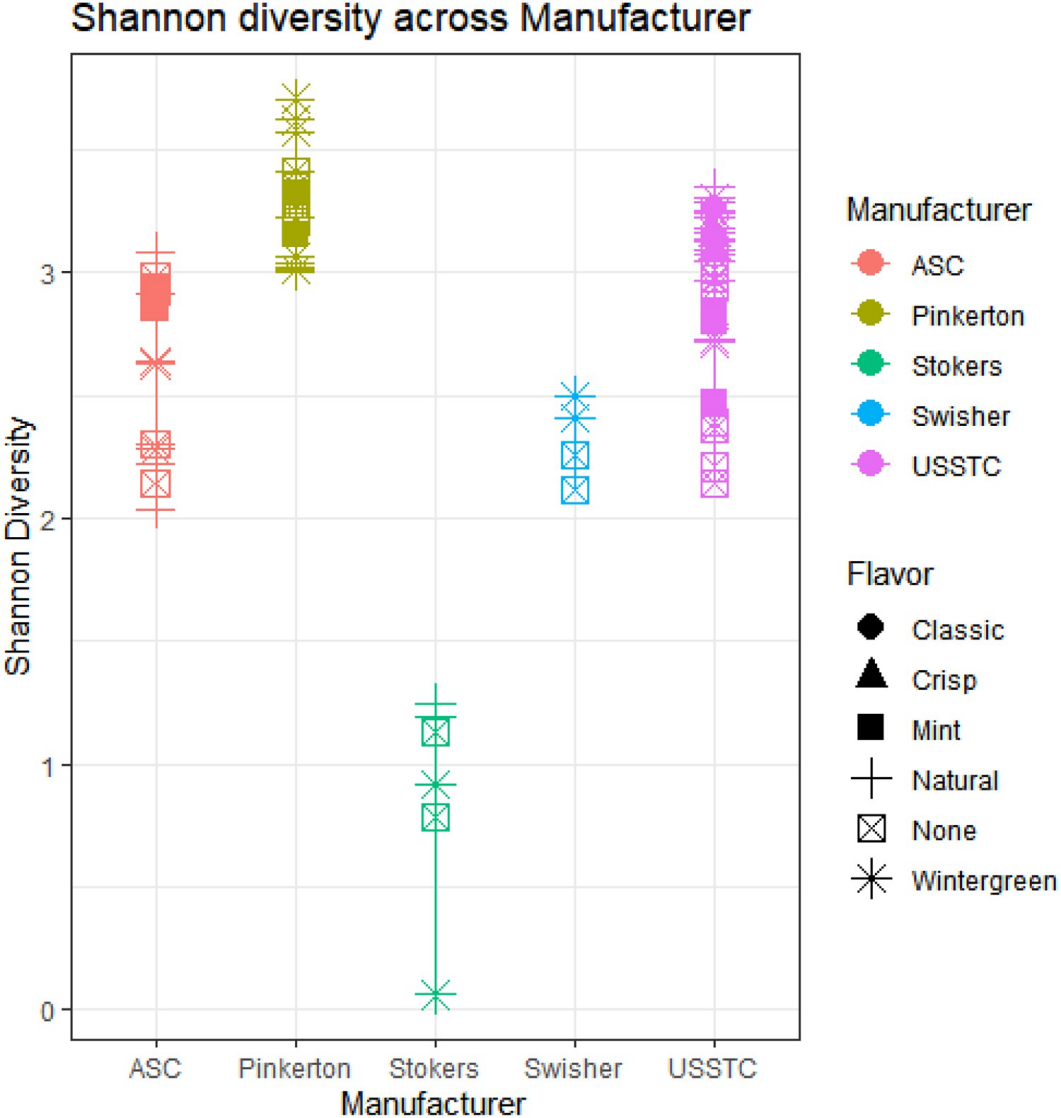
Alpha diversity given by Shannon diversity metric. R package Phyloseq was used to generate a tree based on taxonomy glommed to Genus. Color indicates manufacturer and shape represents flavor type. Sequencing for each sample was conducted in triplicate, with mean of three replicates presented in the figure.

Within-sample product (alpha) diversities, as measured by the Shannon diversity index are shown in Fig 9, which shows that Shannon diversity ranged from less than 0.1 to greater than 3.5. Products from Pinkerton had the greatest microbial diversity within the products we analyzed. U.S. Smokeless Tobacco Company (USSTC), Swisher, and ASC had Shannon diversity values that were similar and less than Pinkerton’s (Fig 9). In contrast, Stoker’s product sampled were differentiated from all other manufacturers by having the lowest Shannon diversity measures, and were heavily dominated by *Bacillus* spp.

When analyzed by manufacturer, the only significant findings were for products manufactured by the American Snuff Company (ASC). We found that *Marinilactibacillus* was negatively correlated with TSNAs. This means that when more of these taxa were present in a product, there was a likelihood of lower values of TSNAs. The *Marinilactibacillus* were the single most abundant organism found in all the samples. *Marinilactibacillus* genus was completely absent in all Stoker’s products and was only found in low amounts in several ASC products. *Tetragenococcus* spp. were found in large amounts in most Pinkerton and some USSTC products.

Microbial community composition also differed substantially between the two variations of Copenhagen Long Cut Straight with different labeling, with sample ‘A’ having a much lower relative abundance of *Tetragenococcus*, a much higher relative abundance of *Atopostipes*, and a somewhat higher abundance of *Marinilactibacillus*.

## Discussion

### Survey of moist snuff

Continued monitoring of smokeless tobacco products is important because its chemical and biological constituents can vary over time, even with products within the same brand. These variations can be due to multiple factors, including sources of tobacco, weather changes (e.g. rainfall, humidity), and changes in farming and manufacturing processes implemented over time [5]. When we compared products previously measured in our lab, we found a reduction in TSNAs for new products identically named. In this study, we noted three Skoal products (Skoal Long Cut Classic, Skoal Long Cut Mint, and Skoal Long Cut Wintergreen), having much lower TSNA values compared to the exact same products in the Richter *et al*., 2008 study [25].

Detailed manufacturing steps of moist snuff products remain as trade secrets. However, communications with regulatory authorities (e.g. FDA contacts), as well as recent comments from industry on proposed TSNAs regulations, suggest that at least some, or perhaps most, manufacturers tailor the microbes used in fermentation in order to minimize TSNAs [17]. This may also explain the patterns observed in the within-sample diversity metric (Shannon diversity), where we found similar values for all products from the same manufacturer.

We found product’s microbiotas segregated readily by manufacturer, but none were notably similar to those previously reported in cigarette tobacco, cured, or aged tobacco leaves [55, 56]. In most of those products, Proteobacteria, not Firmicutes, made up the majority of the taxa identified. At the highest level of taxonomy, most products tested here had a microbiota made up mainly of three genera within Firmicutes. *Marinilactibacillus* spp. were the taxa most likely to be present and were found in 74% of products tested (28/38, Fig 6). The dominance of most of these products by *Marinilactibacillus* contrasts recently characterized little cigar and cigarillo [35, 36] and cigarette microbial communities where this species was not prominent in the products [37].

To date, the majority of previous smokeless tobacco product surveys have not included microbiota data coupled to chemistry data, or have only limited data (e.g. Han, et al.,) [22]. Law, et al., 2016, established correlations between taxa and chemistry but samples in that study were not commercial products [24]. Thus, we attempted to establish associations between chemistry (TSNAs, nicotine, moisture, pH) and specific taxa (OTU abundances) in commercial products.

#### Microbiome data analysis

We conducted separate analyses of 16S relative abundance and absolute abundance data, as well as on the log-ratio scale. We found similar results for both relative and absolute abundance analyses, while results obtained using CLR-transformed data were divergent. This is important as some authors have argued that the only valid analyses of relative abundance data are conducted on the log-ratio scale. While it is true that analyses based on log-ratios are invariant to the (typically unknown) absolute quantity of DNA in a sample, it is telling that the analyses based on relative abundance were in fact consistent with the analyses based on absolute abundances while those based on log-ratios generally reached different conclusions. This finding is consistent with the observation that log-ratio-based analyses test a different hypotheses than those we tested here that were based on differences in absolute abundance [51]. In particular, the log-ratio-based analyses allow changes in the abundances (either relative or absolute) of pairs of taxa to be consistent with the null hypothesis as long as their *ratio* is unchanged. Further, it is known that the choice of a pseudocount can change the conclusions of an analysis [57, 58].

In our analyses, we chose to consider manufacturer as a confounder because we saw that both TSNA levels and microbial composition varied by manufacturer. In future analyses, it may be interesting to determine the extent to which microbial levels mediate the effect of manufacturer on TSNA levels. Flavor was included as a confounder because it appears to have a large affect on product compostion, potentially affecting both the microbes present and in turn the TSNA levels. We also considered nicotine as a potential confounder due to its potential influence as a precursor for NNN. However, nicotine did not have global significance even when manufacturer and flavor were included as confounders. One argument against nicotine confounding associations between microbial taxa and TSNA is the disproportional abundance of nicotine compared to TSNAs in tobacco. Consequently, nicotine should have little direct effect on the amount of TSNAs.

The pH values of these products when TSNAs are being formed are likely to have an effect on TSNA levels, but we did not observe pH to be correlated to TSNAs in these samples, possibly because manufacturers tailor pH for their products after all TSNAs have been generated. Moisture was also tested for significance, but was not found to have significant effects on the microbiota, so it was not considered confounding for the statistical analysis.

*Marinilactibacillus* spp., the most common bacteria found in our samples, has not been well documented to date, with only a few species described and sequenced. None of the species so far identified in the *Marinilactibacillus* genus have an annotated nitrate reductase gene, or have been found to reduce nitrate in culture [59, 60]. It is a possibility that a nitrate reductase will be identified in this genus in the future, but it is more likely that most microbes in the genus do not reduce nitrate.

Most studies of smokeless tobacco products have identified many bacteria known for being halotolerant, including *Marinilactibacillus*. We hypothesize that most bacteria observed in the finished products result from an ongoing selection brought about by the addition of salts and other manufacturing treatments to prevent further TSNA generation. For example, the addition of unknown amounts of sodium chlorate [17], a human toxin if ingested, conventionally used as a weedkiller, was reported by one manufacturer. Sodium chlorate is reported to be added as a competitive inhibitor for nitrate when exposed to nitrate-reducing bacteria, preventing these bacteria from proliferating and generating nitrite in products after packaging. Due to the toxic nature of sodium chlorate, further investigation into the concentrations of sodium chlorate in these products is warranted.

### Comments on individual products

Compared with previous observations of moist snuff tobacco products [25, 53], the chemical measurements of the products in this study were similar to those in the past, but a few trends were noticed including, on average, lower TSNAs than previously observed [18]. Total nicotine concentrations were overall increased, while free (unprotonated) nicotine concentrations were lower than previously observed for products with the same name brands. Differences in chemical concentrations may result from lot to lot variability, influenced by the tobacco source, or from temporal changes during the manufacturing process itself.

Unlike the other products described here, Hawken Wintergreen was previously found to be virtually sterile, by bacterial culturing methods [21]. However, we were able to extract quantifiable amounts of DNA. Although, even when an abundance (by total DNA measurement) of template DNA was used as template material, we were unable to effectively amplify 16S sequences from the extraction, as demonstrated by undetectable amounts of DNA after PCR amplification. While we were able to obtain a useable signal in the bacterial load qPCR, this method uses only a small portion (~125 base pairs) of the ubiquitous 16S rDNA sequence, the measured bacterial load was about 10-fold less than the second lowest product, and almost 100-fold lower than the median measurement of the products. This suggests that either 1) the DNA purified out of Hawken may have been degraded to a great extent, or 2) it originates from Eukaryotic sources. The latter we consider unlikely, based on shotgun metagenomic data of other ST products that showed Eukaryotic microbes in very low abundance in most moist snuff products [6, 61]. Despite Hawken being seemingly bacteria-free as an end product, it had comparable amounts of TSNAs to other moist snuff products, although it was lower in nicotine (by wet weight) than all the other moist snuff products, and much lower in free nicotine. This may further support the idea that the end product microbiota may not represent the microbes that were present when TSNAs were formed [61].

The clear segregation of microbiotas by manufacturer in the products analyzed shows that processing or source of tobacco is more important for a product’s observable communities than added flavor. Further investigation into flavor’s affect on ST microbiota is warranted due to the potential to use flavor to tailor the microbiota away from taxa involved in generation of TSNAs.

### Limitations of the study

We focused on bacterial constituents because Fungi and Eukaryotes have been found to be less prominent in these products. Further, based on previous literature and metagenome studies, bacteria are expected to have larger effects on TSNAs in the manufacturing process [6, 24, 33, 61], than fungi. The microbiota observed may simply reflect fluctuations due to changes in tobacco source due to growing conditions. This change in source material is likely to be linked to the manufacturer-to-manufacturer variability seen in this study. Therefore, one limitation of this study is that it presents samples obtained at a single time point, and from a single vendor. A wider range of sampling may better characterized the product variability and would help identify the stability, or lack thereof, of the microbiota in these products. We also did not consider lot-to-lot variation in these products (except the difference in the two versions of Copenhagen) or the impact of product aging, as the products are not labeled with the manufactured date.

We also acknowledge that this data represents statistical associations only. Experimentation is necessary to investigate whether these taxa may be causal in being responsible for reducing nitrate, and therefore, directly involved in the generation of harmful TSNAs in the production of these products. However, it is very unlikely we could obtain longitudinal samples from a single batch or lot as it goes through the stages of manufacture; even sequential cross-sectional samples are not likely to be made available. As a result, the final-product associations presented here are the only evidence available on any possible relationship between bacteria and chemical composition of ST products. Different product types appear to have different core microbiotas, and it is clear that bacterial constituency in the products we observed do not reflect microbiotas found in raw tobacco [34–36, 38]. Associations identified here may be relevant only for moist snuff and may not be a suitable approach for the specific identification of organisms involved in nitrate reduction, and thus, TSNA generation. Communities observed in off-the-shelf tobacco products, especially moist snuff, which has different consistency from raw tobacco, may not reflect what taxa were present during the active periods of TSNA formation. In the products tested here, many of the bacteria identified are known halotolerant bacteria that may simply reflect such an environment is present in these tobacco products. Many genera, including some of the most abundant in these products such as *Lactobacillus* and *Marinilactibacillus*, do not even include known nitrate reduction genes in their genomes. These observations support a hypothesis that the community may have shifted between the time of TSNA generation and the time we are observing the community in the off-the-shelf product. We suggest further investigation is needed to identify the nitrate-reducing microbes that may be active at earlier time points in the manufacturing process and may be ultimately responsible for TSNA formation. Lastly, the number of products tested here is relatively small for making robust statistical conclusions, where analyzing a larger number could potentially reveal associations inadvertently overlooked in this study.

## Conclusions

This study offers a publicly available large sample set of amplicon sequencing of U.S. domestic moist snuff products. The chemical and microbiota measurements provide a starting and reference point for ongoing explorations of the potential associations between product chemistry and its microbiota. We found a number of taxa were associated with TSNAs, though interpreting these associations in light of their occurrence at the end of a time-dependent process may be problematic. Future studies directed towards samples obtained at earlier time points in the manufacturing process may help greatly reduce the potential confounding variables in this complex system, but such samples would only be available from the manufacturers and so are likely difficult to obtain.

Advancing our knowledge of smokeless tobacco products microbiotas will greatly help in the ability to suggest regulations that may lead to lower toxicant levels. Although lowering exposure for a select, but potent class of carcinogenic chemicals, might not lower overall harm. However, it seems prudent to consider reducing exposures to harmful chemicals in these products, when feasible. Tailoring the bacterial composition by adding species that do not reduce nitrate or increase nitrite assimilation are techniques that have had some demonstrated success in reducing TSNA concentrations, and further research should be encouraged in these areas.

## Supporting information

S1 Fig Free nicotine percentage vs Wintergreen status. (PPTX)

S2 Fig TSNAs by manufacturer. (PPTX)

S3 Fig Multivariate analysis of moist snuff. (PPTX)

S4 Fig Total bacterial load by 16S qPCR. (TIFF)

S1 File Sample PCR setup with indexes. (XLSX)

S2 File Linux and QIIME commands. (TXT)

S3 File Statistical analysis results using R package “LDM”. (TXT)

S4 File (XLSX)

## References

[1] NIH/CDC, editor. Smokeless Tobacco and Public Health: A Global Perspective. Bethesda, MD: US Department of Health and Human Services, Centers for Disease Control and Prevention and National Institutes of Health, National Cancer Institute. NIH Publication No. 14–7983; 2014.

[2] IARC. Smokeless tobacco and some tobacco-specific N-nitrosamines. Monograph. Lyon, France: International Agency for Research of Cancer, 2007.

[3] Creamer MR, Wang TW, Babb S, Cullen KA, Day H, Willis G, et al. Tobacco Product Use and Cessation Indicators Among Adults—United States, 2018. MMWR Morb Mortal Wkly Rep. 2019;68(45):1013–9. Epub 2019/11/15. doi: 10.15585/mmwr.mm6845a2 Journal Editors form for disclosure of potential conflicts of interest. No potential conflicts of interest were disclosed. 31725711 PMC6855510

[4] Brunnemann KD, Prokopczyk B, Djordjevic MV, Hoffmann D. Formation and analysis of tobacco-specific N-nitrosamines. Crit Rev Toxicol. 1996;26(2):121–37. doi: 10.3109/10408449609017926 8688156

[5] Fisher MT, Bennett CB, Hayes A, Kargalioglu Y, Knox BL, Xu DM, et al. Sources of and technical approaches for the abatement of tobacco specific nitrosamine formation in moist smokeless tobacco products. Food Chem Toxicol. 2012;50(3–4):942–8. doi: 10.1016/j.fct.2011.11.035 22142690

[6] Tyx RE, Rivera AJ, Keong LM, Stanfill SB. An exploration of smokeless tobacco product nucleic acids: a combined metagenome and metatranscriptome analysis. Appl Microbiol Biotechnol. 2020;104(2):751–63. doi: 10.1007/s00253-019-10232-3 31820070 PMC6943401

[7] Davis D NM. Tobacco: Production, Chemistry and Technology: Wiley-Blackwell; 1999.

[8] Brunnemann KD QJ, Hoffmann D. Aging of Oral Moist Snuff and the Yields of Tobacco-Specific N-Nitrosamines (TSNA) Progress Report. Prepared for the Massachusetts Tobacco Control Program, Department of Public Health. 2001 June 22, 2001. Report No.

[9] Wang J, Yang H, Shi H, Zhou J, Bai R, Zhang M, et al. Nitrate and Nitrite Promote Formation of Tobacco-Specific Nitrosamines via Nitrogen Oxides Intermediates during Postcured Storage under Warm Temperature. Journal of Chemistry. 2017;2017:6135215. doi: 10.1155/2017/6135215

[10] Rowe JJ, Ubbinkkok T, Molenaar D, Konings WN, Driessen AJM. Nark Is a Nitrite-Extrusion System Involved in Anaerobic Nitrate Respiration by Escherichia-Coli. Mol Microbiol. 1994;12(4):579–86. doi: 10.1111/j.1365-2958.1994.tb01044.x 7934881

[11] Sohaskey CD, Wayne LG. Role of narK2X and narGHJI in hypoxic upregulation of nitrate reduction by Mycobacterium tuberculosis. J Bacteriol. 2003;185(24):7247–56. doi: 10.1128/JB.185.24.7247-7256.2003 14645286 PMC296237

[12] Hoffmann D, Adams JD, Lisk D, Fisenne I, Brunnemann KD. Toxic and carcinogenic agents in dry and moist snuff. J Natl Cancer Inst. 1987;79(6):1281–6. . 3480379

[13] Hoffmann D, Harley NH, Fisenne I, Adams JD, Brunnemann KD. Carcinogenic agents in snuff. J Natl Cancer Inst. 1986;76(3):435–7. . 3456461

[14] IARC. Agents Classified by the IARC Monographs, Vol. 1–107.2013. http://monographs.iarc.fr/ENG/Classification/ClassificationsGroupOrder.pdf.

[15] Tanaka K, Hayatsu T, Negishi T, Hayatsu H. Inhibition of N-nitrosation of secondary amines in vitro by tea extracts and catechins. Mutat Res-Gen Tox En. 1998;412(1):91–8. doi: 10.1016/s1383-5718(97)00178-2 9508368

[16] Wei X, Deng X, Cai D, Ji Z, Wang C, Yu J, et al. Decreased tobacco-specific nitrosamines by microbial treatment with Bacillus amyloliquefaciens DA9 during the air-curing process of burley tobacco. J Agric Food Chem. 2014;62(52):12701–6. doi: 10.1021/jf504084z . 25514373

[17] Altria: Murillo JL. Altria Comments to Proposed FDA Rule: Dock No. FDA-2016-N-2527. Division of Dockets Management—Food and Drug Administration2017.

[18] Oldham MJ, Lion KE 3rd, Phillips DJ, Morton MJ, Lusso MF, Harris EA, et al. Variability of TSNA in U.S. Tobacco and Moist Smokeless Tobacco Products. Toxicology reports. 2020;7:752–8. Epub 2020/07/03. doi: 10.1016/j.toxrep.2020.05.008 32612935 PMC7317684

[19] Monika S, Dineshkumar T, Priyadharini S, Niveditha T, Sk P, Rajkumar K. Smokeless Tobacco Products (STPs) Harbour Bacterial Populations with Potential for Oral Carcinogenicity. Asian Pacific journal of cancer prevention: APJCP. 2020;21(3):815–24. Epub 2020/03/28. doi: 10.31557/APJCP.2020.21.3.815 . 32212812 PMC7437332

[20] Tyx RE, Stanfill SB, Keong LM, Rivera AJ, Satten GA, Watson CH. Characterization of Bacterial Communities in Selected Smokeless Tobacco Products Using 16S rDNA Analysis. PLoS One. 2016;11(1):e0146939. doi: 10.1371/journal.pone.0146939 26784944 PMC4718623

[21] Smyth EM, Kulkarni P, Claye E, Stanfill S, Tyx R, Maddox C, et al. Smokeless tobacco products harbor diverse bacterial microbiota that differ across products and brands. Appl Microbiol Biotechnol. 2017. doi: 10.1007/s00253-017-8282-9 . 28432442 PMC5520664

[22] Han J, Sanad YM, Deck J, Sutherland JB, Li Z, Walters MJ, et al. Bacterial Populations Associated with Smokeless Tobacco Products. Appl Environ Microbiol. 2016;82(20):6273–83. Epub 2016/08/28. doi: 10.1128/AEM.01612-16 27565615 PMC5068160

[23] Al-Hebshi NN, Alharbi FA, Mahri M, Chen T. Differences in the Bacteriome of Smokeless Tobacco Products with Different Oral Carcinogenicity: Compositional and Predicted Functional Analysis. Genes. 2017;8(4). Epub 2017/03/24. doi: 10.3390/genes8040106 28333122 PMC5406853

[24] Law AD, Fisher C, Jack A, Moe LA. Tobacco, Microbes, and Carcinogens: Correlation Between Tobacco Cure Conditions, Tobacco-Specific Nitrosamine Content, and Cured Leaf Microbial Community. Microb Ecol. 2016;72(1):120–9. Epub 2016/03/30. doi: 10.1007/s00248-016-0754-4 . 27023797

[25] Richter P, Hodge K, Stanfill S, Zhang L, Watson C. Surveillance of moist snuff: total nicotine, moisture, pH, un-ionized nicotine, and tobacco-specific nitrosamines. Nicotine Tob Res. 2008;10(11):1645–52. 10.1080/14622200802412937 . 18988077

[26] Hearn BA, Renner CC, Ding YS, Vaughan-Watson C, Stanfill SB, Zhang L, et al. Chemical Analysis of Alaskan Iq’mik Smokeless Tobacco. Nicotine Tob Res. 2013. doi: 10.1093/ntr/nts270 . 23288872 PMC7814975

[27] Stanfill SB, Connolly GN, Zhang L, Jia LT, Henningfield JE, Richter P, et al. Global surveillance of oral tobacco products: total nicotine, unionised nicotine and tobacco-specific N-nitrosamines. Tob Control. 2011;20(3):e2. 10.1136/tc.2010.037465 . 21109685

[28] Stepanov I, Biener L, Knezevich A, Nyman AL, Bliss R, Jensen J, et al. Monitoring tobacco-specific N-nitrosamines and nicotine in novel Marlboro and Camel smokeless tobacco products: findings from Round 1 of the New Product Watch. Nicotine Tob Res. 2012;14(3):274–81. doi: 10.1093/ntr/ntr209 22039075 PMC3281237

[29] Stepanov I, Jensen J, Biener L, Bliss RL, Hecht SS, Hatsukami DK. Increased pouch sizes and resulting changes in the amounts of nicotine and tobacco-specific N-nitrosamines in single pouches of Camel Snus and Marlboro Snus. Nicotine Tob Res. 2012;14(10):1241–5. doi: 10.1093/ntr/ntr292 22259150 PMC3457708

[30] Stepanov I, Knezevich A, Zhang L, Watson CH, Hatsukami DK, Hecht SS. Carcinogenic tobacco-specific N-nitrosamines in US cigarettes: three decades of remarkable neglect by the tobacco industry. Tob Control. 2012;21(1):44–8. doi: 10.1136/tc.2010.042192 21602537 PMC3572908

[31] Stepanov I, Yershova K, Carmella S, Upadhyaya P, Hecht SS. Levels of (S)-N’-Nitrosonornicotine in U.S. Tobacco Products. Nicotine Tob Res. 2012. doi: 10.1093/ntr/nts249 . 23212437 PMC3682840

[32] Lawler TS, Stanfill SB, Zhang L, Ashley DL, Watson CH. Chemical characterization of domestic oral tobacco products: total nicotine, pH, unprotonated nicotine and tobacco-specific N-nitrosamines. Food Chem Toxicol. 2013;57:380–6. doi: 10.1016/j.fct.2013.03.011 . 23517910 PMC5659123

[33] Di Giacomo M, Paolino M, Silvestro D, Vigliotta G, Imperi F, Visca P, et al. Microbial community structure and dynamics of dark fire-cured tobacco fermentation. Appl Environ Microb. 2007;73(3):825–37. doi: 10.1128/AEM.02378-06 17142368 PMC1800767

[34] Chopyk J, Chattopadhyay S, Kulkarni P, Smyth EM, Hittle LE, Paulson JN, et al. Temporal Variations in Cigarette Tobacco Bacterial Community Composition and Tobacco-Specific Nitrosamine Content Are Influenced by Brand and Storage Conditions. Front Microbiol. 2017;8:358. doi: 10.3389/fmicb.2017.00358 28326071 PMC5339245

[35] Smyth EM, Chattopadhyay S, Babik K, Reid M, Chopyk J, Malayil L, et al. The Bacterial Communities of Little Cigars and Cigarillos Are Dynamic Over Time and Varying Storage Conditions. Front Microbiol. 2019;10:2371. Epub 2019/11/12. doi: 10.3389/fmicb.2019.02371 31708882 PMC6824217

[36] Chattopadhyay S, Smyth EM, Kulkarni P, Babik KR, Reid M, Hittle LE, et al. Little cigars and cigarillos harbor diverse bacterial communities that differ between the tobacco and the wrapper. PLoS One. 2019;14(2):e0211705. Epub 2019/02/23. doi: 10.1371/journal.pone.0211705 30794551 PMC6386278

[37] Chopyk J, Chattopadhyay S, Kulkarni P, Claye E, Babik KR, Reid MC, et al. Mentholation affects the cigarette microbiota by selecting for bacteria resistant to harsh environmental conditions and selecting against potential bacterial pathogens. Microbiome. 2017;5(1):22. doi: 10.1186/s40168-017-0235-0 28202080 PMC5312438

[38] Sapkota AR, Berger S, Vogel TM. Human Pathogens Abundant in the Bacterial Metagenome of Cigarettes. Environ Health Persp. 2010;118(3):351–6. doi: 10.1289/ehp.0901201 20064769 PMC2854762

[39] Rutqvist LE, Curvall M, Hassler T, Ringberger T, Wahlberg I. Swedish snus and the GothiaTek (R) standard. Harm Reduct J. 2011;8. Artn 11 doi: 10.1186/1477-7517-8-11 21575206 PMC3119032

[40] Ashley DL, Pankow JF, Tavakoli AD, Watson CH. Approaches, challenges, and experience in assessing free nicotine. Handbook of experimental pharmacology. 2009;(192):437–56. Epub 2009/02/03. doi: 10.1007/978-3-540-69248-5_15 . 19184658

[41] Federal_Register. Annual Submission of the Quantity of Nicotine Contained in Smokeless Tobacco Products Manufactured, Imported, or Packaged in the United States. pp. 14085–14096 FR Doc 99–7022 1999. p. pp. 14085–96. 10558405

[42] Stanfill SB, Jia LT, Ashley DJ, Watson CH. Rapid and chemically selective nicotine quantification in smokeless tobacco products using GC-MS. J Chromatogr Sci. 2009;47(10):902–9. doi: 10.1093/chromsci/47.10.902 . 19930803

[43] Nelson MC, Morrison HG, Benjamino J, Grim SL, Graf J. Analysis, optimization and verification of Illumina-generated 16S rRNA gene amplicon surveys. PLoS One. 2014;9(4):e94249. Epub 2014/04/12. doi: 10.1371/journal.pone.0094249 24722003 PMC3983156

[44] Fadrosh DW, Ma B, Gajer P, Sengamalay N, Ott S, Brotman RM, et al. An improved dual-indexing approach for multiplexed 16S rRNA gene sequencing on the Illumina MiSeq platform. Microbiome. 2014;2(1):6. Epub 2014/02/25. doi: 10.1186/2049-2618-2-6 24558975 PMC3940169

[45] Kozich JJ, Westcott SL, Baxter NT, Highlander SK, Schloss PD. Development of a dual-index sequencing strategy and curation pipeline for analyzing amplicon sequence data on the MiSeq Illumina sequencing platform. Appl Environ Microbiol. 2013;79(17):5112–20. Epub 2013/06/25. doi: 10.1128/AEM.01043-13 23793624 PMC3753973

[46] Bolyen E, Rideout JR, Dillon MR, Bokulich NA, Abnet CC, Al-Ghalith GA, et al. Reproducible, interactive, scalable and extensible microbiome data science using QIIME 2. Nature biotechnology. 2019;37(8):852–7. Epub 2019/07/26. doi: 10.1038/s41587-019-0209-9 . 31341288 PMC7015180

[47] Callahan BJ, McMurdie PJ, Rosen MJ, Han AW, Johnson AJ, Holmes SP. DADA2: High-resolution sample inference from Illumina amplicon data. Nat Methods. 2016;13(7):581–3. doi: 10.1038/nmeth.3869 27214047 PMC4927377

[48] Pruesse E, Quast C, Knittel K, Fuchs BM, Ludwig W, Peplies, et al. SILVA: a comprehensive online resource for quality checked and aligned ribosomal RNA sequence data compatible with ARB. Nucleic Acids Res. 2007;35. doi: 10.1093/nar/gkm864 17947321 PMC2175337

[49] Quast C, Pruesse E, Yilmaz P, Gerken J, Schweer T, Yarza P, et al. The SILVA ribosomal RNA gene database project: improved data processing and web-based tools. Nucleic Acids Res. 2013;41(Database issue):D590–6. doi: 10.1093/nar/gks1219 23193283 PMC3531112

[50] McMurdie PJ, Holmes S. phyloseq: an R package for reproducible interactive analysis and graphics of microbiome census data. PLoS One. 2013;8(4):e61217. doi: 10.1371/journal.pone.0061217 23630581 PMC3632530

[51] Hu YJ, Satten GA. Testing hypotheses about the microbiome using the linear decomposition model (LDM). Bioinformatics. 2020;36(14):4106–15. Epub 2020/04/22. doi: 10.1093/bioinformatics/btaa260 . 32315393 PMC8453243

[52] Hawinkel S, Mattiello F, Bijnens L, Thas O. A broken promise: microbiome differential abundance methods do not control the false discovery rate. Brief Bioinform. 2019;20(1):210–21. Epub 2017/10/03. doi: 10.1093/bib/bbx104 . 28968702

[53] Stepanov I, Jensen J, Hatsukami D, Hecht SS. New and traditional smokeless tobacco: comparison of toxicant and carcinogen levels. Nicotine Tob Res. 2008;10(12):1773–82. doi: 10.1080/14622200802443544 19023828 PMC2892835

[54] Richter P, Spierto FW. Surveillance of smokeless tobacco nicotine, pH, moisture, and unprotonated nicotine content. Nicotine Tob Res. 2003;5(6):885–9. Epub 2003/12/12. doi: 10.1080/14622200310001614647 . 14668072

[55] Su C, Gu W, Zhe W, Zhang KQ, Duan YQ, Yang JK. Diversity and phylogeny of bacteria on Zimbabwe tobacco leaves estimated by 16S rRNA sequence analysis. Appl Microbiol Biotechnol. 2011;92(5):1033–44. doi: 10.1007/s00253-011-3367-3 21660545

[56] Zhou J, Yu L, Zhang J, Zhang X, Xue Y, Liu J, et al. Characterization of the core microbiome in tobacco leaves during aging. MicrobiologyOpen. 2020;9(3):e984. Epub 2020/01/02. doi: 10.1002/mbo3.984 31893578 PMC7066457

[57] Costea PI, Zeller G, Sunagawa S, Bork P. A fair comparison. Nat Methods. 2014;11(4):359. Epub 2014/04/01. doi: 10.1038/nmeth.2897 . 24681719

[58] Paulson JN, Bravo HC, Pop M. Reply to: "a fair comparison". Nat Methods. 2014;11(4):359–60. Epub 2014/04/01. doi: 10.1038/nmeth.2898 . 24681718

[59] Toffin L, Zink K, Kato C, Pignet P, Bidault A, Bienvenu N, et al. Marinilactibacillus piezotolerans sp. nov., a novel marine lactic acid bacterium isolated from deep sub-seafloor sediment of the Nankai Trough. Int J Syst Evol Microbiol. 2005;55(Pt 1):345–51. Epub 2005/01/18. doi: 10.1099/ijs.0.63236-0 . 15653899

[60] Ishikawa M, Nakajima K, Yanagi M, Yamamoto Y, Yamasato K. Marinilactibacillus psychrotolerans gen. nov., sp. nov., a halophilic and alkaliphilic marine lactic acid bacterium isolated from marine organisms in temperate and subtropical areas of Japan. Int J Syst Evol Microbiol. 2003;53(Pt 3):711–20. Epub 2003/06/17. doi: 10.1099/ijs.0.02446-0 . 12807191

[61] Rivera AJ, Tyx RE, Keong LM, Stanfill SB, Watson CH. Microbial communities and gene contributions in smokeless tobacco products. Appl Microbiol Biotechnol. 2020;104(24):10613–29. Epub 2020/11/13. doi: 10.1007/s00253-020-10999-w . 33180172 PMC7849185

