# Supplementary figures and images for: Associations between microbial communities and key chemical constituents in U.S. domestic moist snuff
Source: PLoS One. 2022 May 4;17(5):e0267104. doi: 10.1371/journal.pone.0267104 (PMC9067656; doi:10.1371/journal.pone.0267104)

## Slide 1
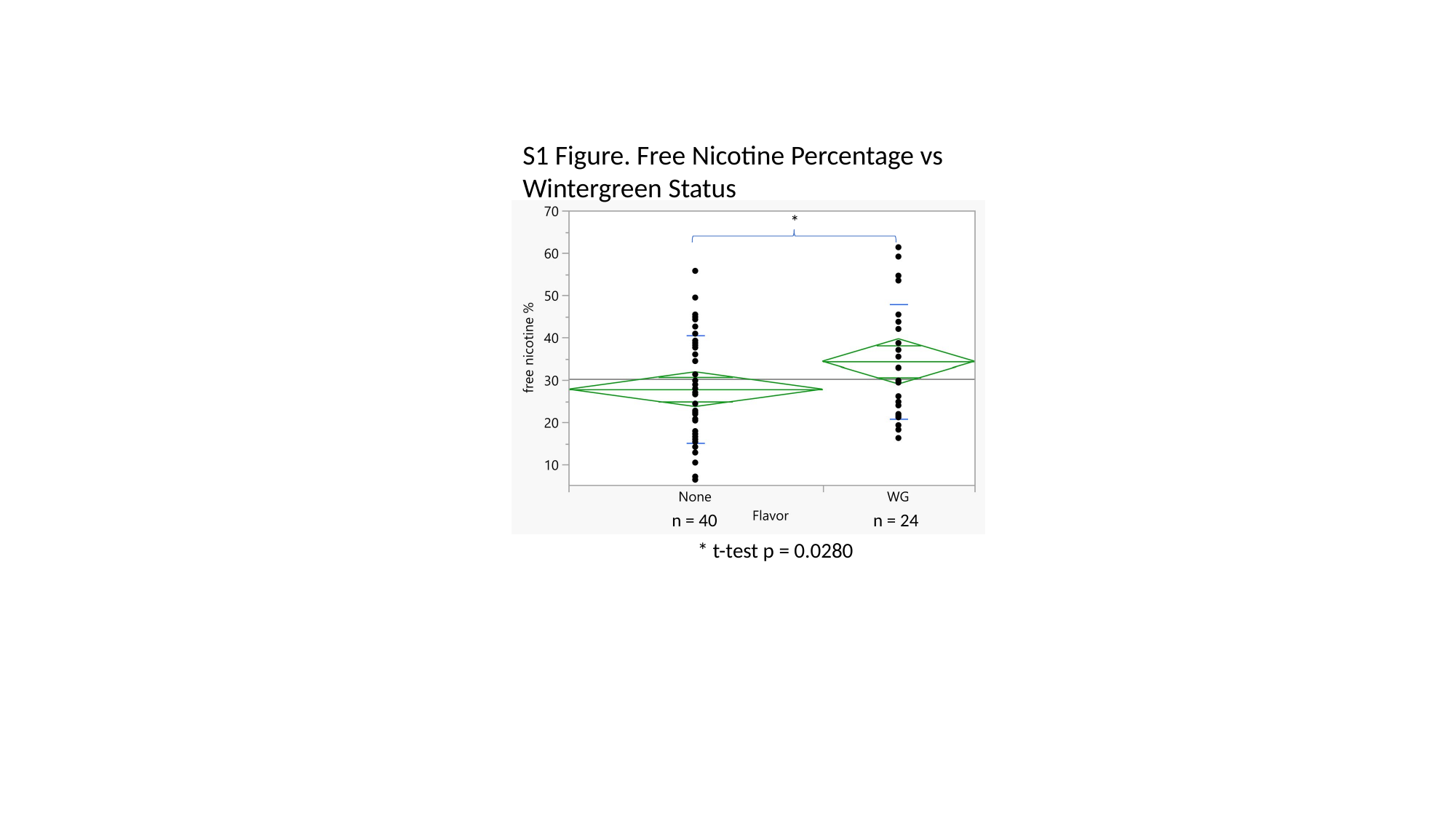

S1 Figure. Free Nicotine Percentage vs Wintergreen Status
*
n = 40
n = 24
* t-test p = 0.0280

Supplement: S1 Fig — (PPTX) [file pone.0267104.s001.pptx]

## Slide 1
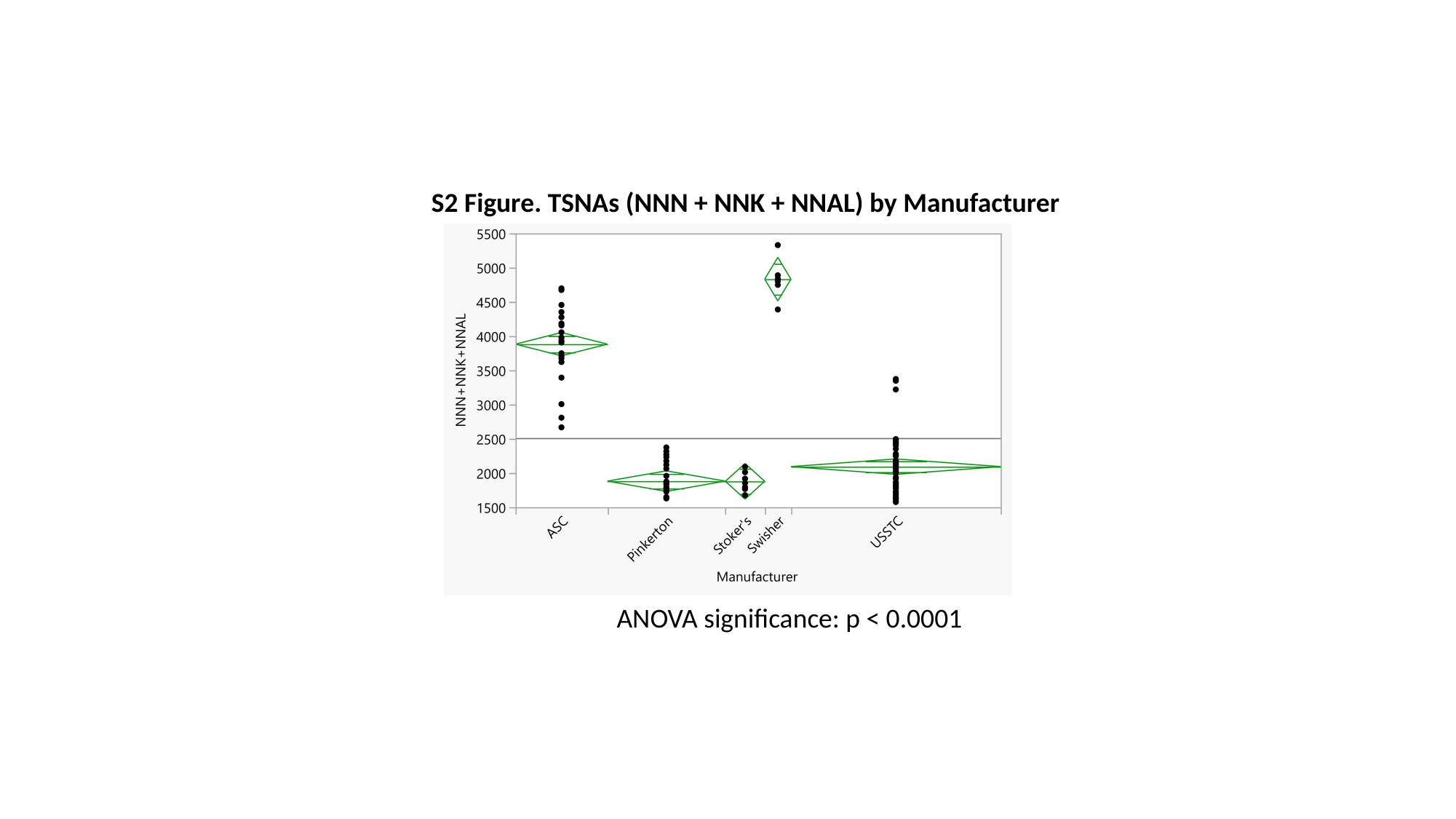

S2 Figure. TSNAs (NNN + NNK + NNAL) by Manufacturer
ANOVA significance: p < 0.0001

Supplement: S2 Fig — (PPTX) [file pone.0267104.s002.pptx]

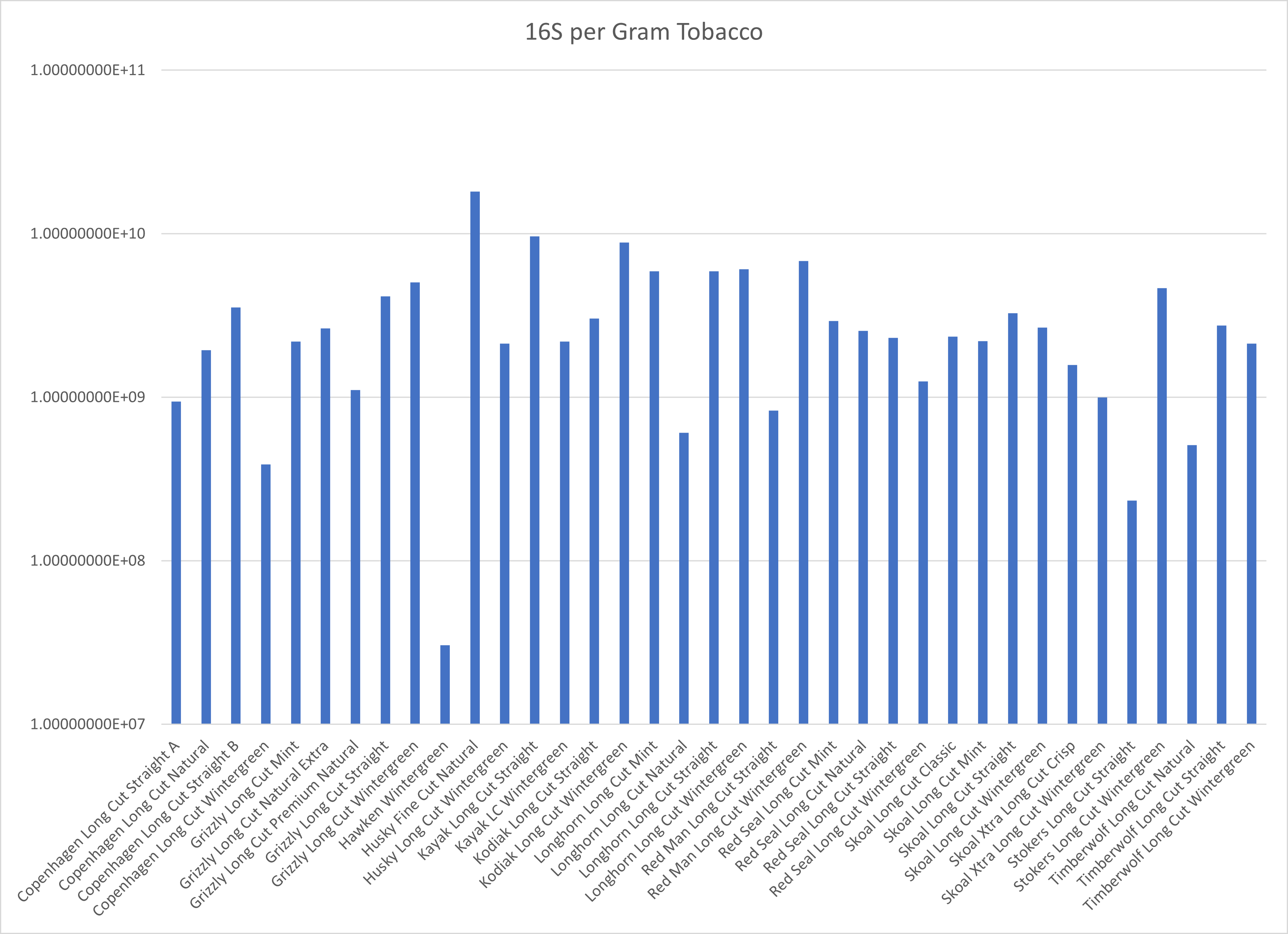

Supplement: S4 Fig — (TIFF) [file pone.0267104.s004.tiff]
