# Supplementary material for: Associations between microbial communities and key chemical constituents in U.S. domestic moist snuff
Source: PLoS One. 2022 May 4;17(5):e0267104. doi: 10.1371/journal.pone.0267104 (PMC9067656; doi:10.1371/journal.pone.0267104)
Supplement: S3 Fig — (PPTX) [file pone.0267104.s003.pptx]

## Slide 1
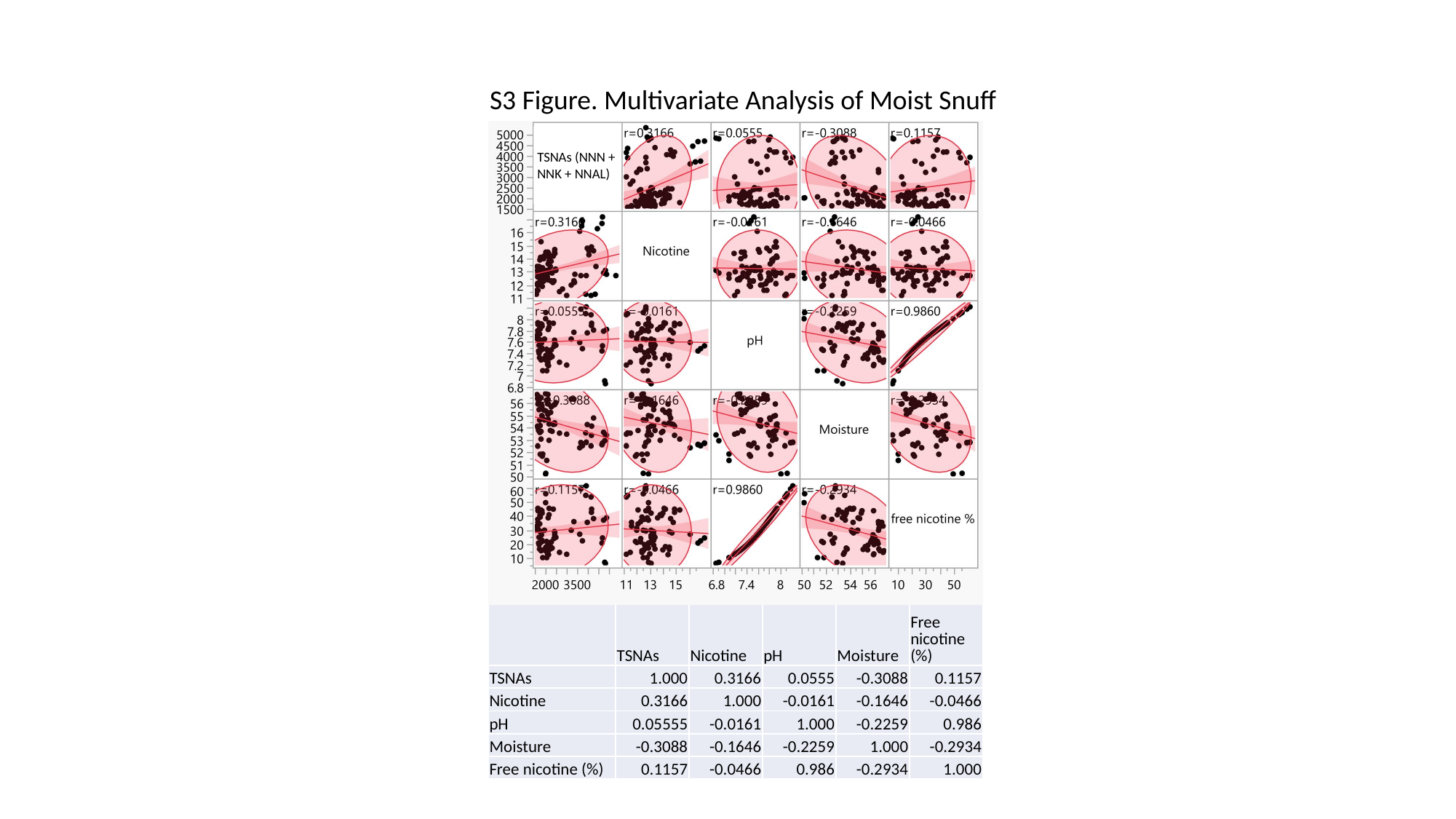

S3 Figure. Multivariate Analysis of Moist Snuff
TSNAs (NNN + NNK + NNAL)
| | TSNAs | Nicotine | pH | Moisture | Free nicotine (%) |
| --- | --- | --- | --- | --- | --- |
| TSNAs | 1.000 | 0.3166 | 0.0555 | -0.3088 | 0.1157 |
| Nicotine | 0.3166 | 1.000 | -0.0161 | -0.1646 | -0.0466 |
| pH | 0.05555 | -0.0161 | 1.000 | -0.2259 | 0.986 |
| Moisture | -0.3088 | -0.1646 | -0.2259 | 1.000 | -0.2934 |
| Free nicotine (%) | 0.1157 | -0.0466 | 0.986 | -0.2934 | 1.000 |
